# Clonal evolution of hematopoietic stem cells after autologous stem cell transplantation

**DOI:** 10.1038/s41588-025-02235-w

**Published:** 2025-07-01

**Authors:** Hidetaka Uryu, Koichi Saeki, Hiroshi Haeno, Chiraag Deepak Kapadia, Ken Furudate, Jyoti Nangalia, Michael Spencer Chapman, Linda Zhang, Jennifer Padilla, Li Zhao, Joanne I. Hsu, Chong Zhao, Shujuan Chen, Tomoyuki Tanaka, Zongrui Li, Satoko Ogata, Sarah Hanache, Hui Yang, Courtney DiNardo, Naval Daver, Naveen Pemmaraju, Nitin Jain, Farhad Ravandi, Jianhua Zhang, Xingzhi Song, Erika Thompson, Hongli Tang, Latasha Little, Curtis Gumbs, Robert Z. Orlowski, Muzaffar Qazilbash, Kapil Bhalla, Simona Colla, Hagop Kantarjian, Rashmi Kanagal-Shamanna, Carlos Bueso-Ramos, Daisuke Nakada, Gheath Al-Atrash, Jeffery Molldrem, P. Andrew Futreal, Elizabeth Shpall, Margaret Goodell, Guillermo Garcia-Manero, Koichi Takahashi

**Affiliations:** 1 Department of Leukemia, The University of Texas MD Anderson Cancer Center, Houston, TX USA; 2 Tokyo University of Science, Division of Integrated Research, Research Institute for Biomedical Sciences, Noda, Japan; 3 Department of Molecular and Cellular Biology, Baylor College of Medicine, Houston, TX USA; 4 Wellcome Sanger Institute, Wellcome Genome Campus, Hinxton, UK; 5 Barts Cancer Institute, Queen Mary University of London, London, UK; 6 Department of Genomic Medicine, The University of Texas MD Anderson Cancer Center, Houston, TX USA; 7 Department of Medicine, Dana–Farber Cancer Institute, Harvard Medical School, Boston, MA USA; 8 Department of Hematopathology, The University of Texas MD Anderson Cancer Center, Houston, TX USA; 9 Department of Genetics, The University of Texas MD Anderson Cancer Center, Houston, TX USA; 10 Department of Lymphoma and Myeloma, The University of Texas MD Anderson Cancer Center, Houston, TX USA; 11 Department of Experimental Therapeutics, The University of Texas MD Anderson Cancer Center, Houston, TX USA; 12 Department of Stem Cell Transplantation and Cellular Therapy, The University of Texas MD Anderson Cancer Center, Houston, TX USA; 13 Department of Molecular and Human Genetics, Baylor College of Medicine, Houston, TX USA

**Keywords:** Genetics research, Acute myeloid leukaemia

## Abstract

The impact of exogenous stressors, such as cancer chemotherapies, on the genomic integrity and clonal dynamics of normal hematopoiesis is not well defined. We conducted whole-genome sequencing on 1,276 single-cell-derived hematopoietic stem and progenitor cell (HSPC) colonies from ten patients with multiple myeloma treated with chemotherapies and six normal donors. Melphalan treatment significantly increased the mutational burden, producing a distinctive mutation signature, whereas other chemotherapeutic agents had minimal effects. Consequently, the clonal diversity and architecture of post-treatment HSPCs resemble those observed in normal elderly individuals, particularly through the progression of oligoclonal hematopoiesis, thereby suggesting that chemotherapy accelerates clonal aging. Integrated phylogenetic analysis of matched therapy-related myeloid neoplasm samples traced their clonal origin to a single-HSPC clone among multiple competing clones, supporting a model of oligoclonal to monoclonal transformation. These findings underscore the need for further systematic research on the long-term hematological consequences of cancer chemotherapy.

## Abstract (web summary)

Analysis of whole-genome sequencing data from hematopoietic stem and progenitor cells taken from patients with myeloma shows how treatment shapes clonal architecture and sheds light on the evolution of treatment-related myeloid neoplasms.

## Main

The acquisition of somatic DNA mutations is a hallmark of aging and contributes to the development of various age-associated human diseases, including cancer^1^. In hematopoietic stem cells (HSCs), mutation acquisition follows a remarkably linear trajectory over time, with individual HSCs acquiring approximately 16–25 mutations per year^2–6^. Certain somatic mutations enhance cell fitness, promoting their selection as driver mutations. This results in clonal expansion of cells harboring these driver mutations, known as clonal hematopoiesis (CH)^7,8^. CH progression contributes to the age-related decline of clonal diversity in HSCs and ultimately to the development of hematologic malignancies^2,9^.

External stressors or carcinogens can modulate mutation rates and cellular dynamics in human tissues. Factors such as ultraviolet (UV) light, tobacco smoking and alcohol intake increase somatic mutations in tissues like skin, lung and esophagus^10–14^. Furthermore, these extrinsic factors alter the fitness landscape of cellular ecosystems, enabling the expansion of cells with mutations that confer a fitness advantage under such conditions^10,13–15^.

The hematopoietic system is also exposed to various external stressors, including cytotoxic chemotherapy, which in rare instances can lead to the development of therapy-related myeloid neoplasms (t-MNs)^16^. DNA sequencing studies in chemotherapy-treated patients with cancer revealed distinct CH mutational profiles relative to the general population, with enrichment of DNA-damage response (DDR) pathway gene mutations, including *TP53*, *PPM1D* and *CHEK2* (refs. ^17,18^). In animal models, DNA-damaging chemotherapy has been demonstrated to promote the selective expansion of HSCs with *TP53* and *PPM1D* mutations^19–21^.

Although prior studies revealed the link between chemotherapy exposure and clonal expansion of specific driver mutations, how chemotherapy influences overall population dynamics and individual human HSC genomes remains unclear. Moreover, how these effects contribute to treatment-induced malignancy remains unclear. Here we employed single-cell colony whole-genome sequencing (WGS) to investigate chemotherapy’s impact on the genome and population dynamics of normal HSCs, using peripheral blood stem cells (PBSCs) from patients previously treated with chemotherapy. By analyzing the matched t-MN genome, we also elucidated the evolutionary history of t-MN development arising from normal HSCs.

## Results

### WGS of single-HSPC colonies from chemo-treated patients

**Fig. 1:**
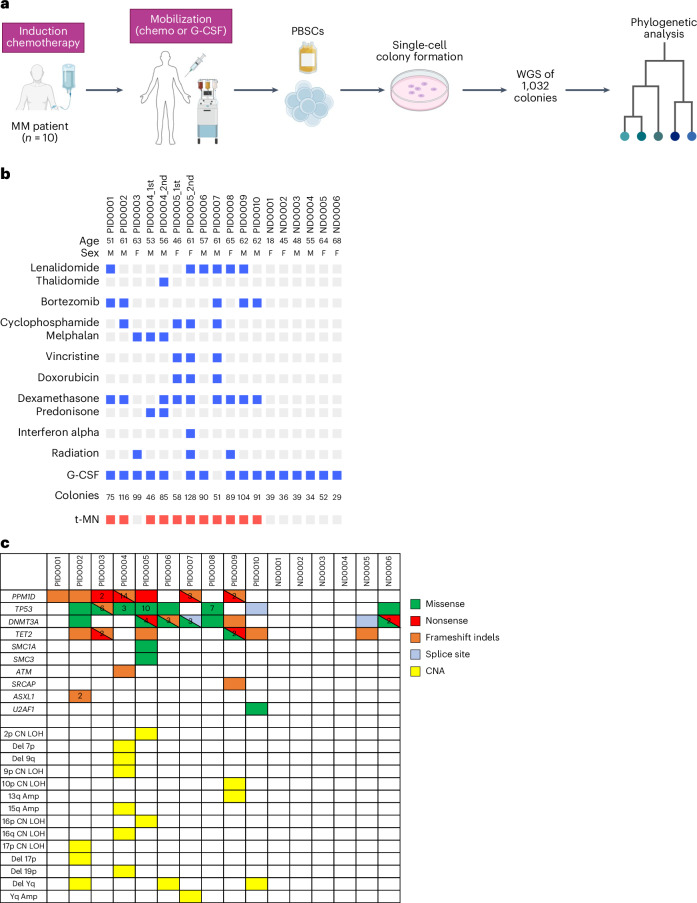
Overview of study design and cohort. **a**, Diagram illustrating the experimental workflow. **b**, Summarized PID and ND demographics, specific chemotherapeutic agents encountered by the analyzed HSPCs, the quantity of colonies evaluated and the occurrence of t-MN. Bright red boxes denote patients who developed t-MNs. **c**, Oncoplot of driver mutations and CNAs identified in at least one colony per patient and normal donor. The numeric value within each box represents the count of distinct mutations identified within the same gene. PID, patient ID; ND, normal donor. Panel **a** created with BioRender.com.

**Extended Data Fig. 1:**
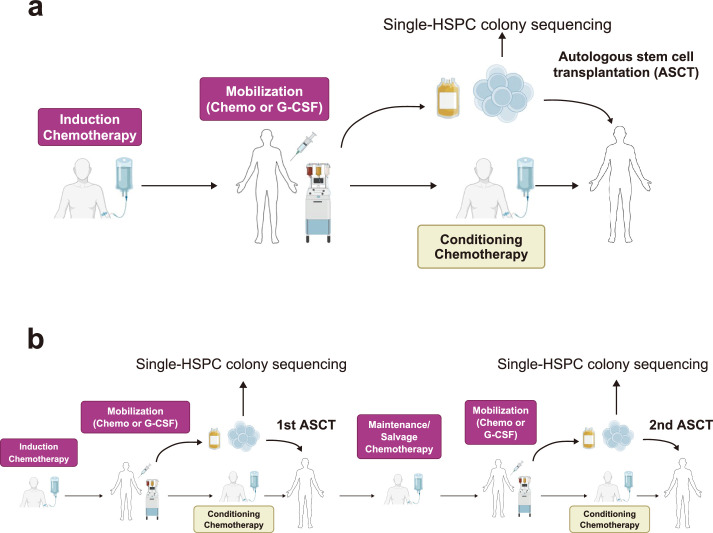
Visual summary of chemotherapy exposure for mobilized PBSCs. **a**, Diagram depicting the chemotherapy exposure of PBSCs from patients undergoing a single ASCT, which includes agents administered during induction therapy and any mobilization regimen. **b**, For patients receiving two ASCTs, this figure outlines the additional exposure of second-timepoint PBSCs to chemotherapies administered during induction, the first mobilization, any maintenance or salvage therapies post-first ASCT, and the mobilization for the second ASCT. Notably, in both scenarios, PBSCs do not encounter chemotherapies applied during the conditioning phase of the transplant. PBSCs analyzed with single-HSPC colony sequencing are specified. The schematics in **a** and **b** are created with BioRender.com.

To study the impact of chemotherapy on the genome of hematopoietic stem and progenitor cells (HSPCs), we analyzed mobilized PBSCs collected from ten patients with multiple myeloma (MM) aged between 46 and 65 years (Fig. 1a). These patients were previously treated with various induction chemotherapies and underwent PBSC collection before autologous stem cell transplantation (ASCT). Two patients underwent ASCT twice with a different PBSC collection at an interval of 3 years and 15 years, respectively, and PBSCs collected from both timepoints were studied. To understand the clonal evolution from normal HSPCs to t-MNs, we enriched the cohort with patients who subsequently developed t-MNs. PBSCs from six normal donors (aged = 18–68 years) with no chemotherapy history were utilized as controls. Table 1 describes the detailed clinical characteristics of the ten patients. As PBSCs are collected before ASCT, cells are exposed to chemotherapies used for induction and mobilization (if chemotherapy is used), but not to the chemotherapies used for transplant conditioning because PBSCs are infused after completing conditioning chemotherapy (Extended Data Fig. 1a). For PBSCs collected at the second ASCT, chemotherapy exposure also includes maintenance or salvage therapies given after the first ASCT and mobilization therapy before the second PBSC collection, in addition to the exposure for the first ASCT (Extended Data Fig. 1b). Of note, in both scenarios, PBSCs are not exposed to high-dose conditioning chemotherapies. Therefore, prior therapy exposures for HSPCs included melphalan (*n* = 2), cyclophosphamide (*n* = 3), doxorubicin (*n* = 2), vincristine (*n* = 2), lenalidomide or thalidomide (*n* = 7), bortezomib (*n* = 5), radiation (*n* = 2) and interferon alpha (*n* = 1; Fig. 1b). This variation in the prior therapy allowed us to study the effect of both conventional cytotoxic chemotherapies (melphalan, cyclophosphamide, doxorubicin and vincristine) and noncytotoxic therapies (lenalidomide, thalidomide and bortezomib) on HSPC genomes.

**Table 1:** Clinical characteristics of ten patients with MM

Patient ID	Sex	Induction chemotherapy	Mobilization	Conditioning	Age at first PBSCH	Chemotherapy after first auto	Mobilization for second auto	Conditioning for second auto	Age at second PBSCH	Number of colonies (first/second)	t-MNs (intervals from last auto)
PID0001	Male	Lenalidomide/bortezomib/dexamethasone	G-CSF	Melphalan	51	–	–	–	–	75	t-MDS (3 years)
PID0002	Male	Cyclophosphamide/bortezomib/dexamethasone	G-CSF	Melphalan	61	–	–	–	–	116	t-MDS (1 year)
PID0003	Female	Melphalan + XRT	G-CSF	Melphalan + lenalidomide	63	–	–	–	–	99	–
PID0004	Male	Melphalan + prednisone	G-CSF	Topotecan, cyclophosphamide, melphalan	53	Thalidomide, dexamethasone	G-CSF	Melphalan	56	133 (46/87)	t-MDS (4 years)
PID0005	Female	Vincristine, doxorubicin, dexamethasone (VAD)	Cyclophosphamide	Thiotepa, busulfan, cyclophosphamide	46	IFN-α, XRT, lenalidomide	G-CSF	Melphalan, lenalidomide	61	177 (49/128)	t-MDS (6 years)
PID0006	Male	Lenalidomide	G-CSF	Melphalan	57	–	–	–	–	90	t-MDS (7 years)
PID0007	Male	Lenalidomide, bortezomib, dexamethasone	CVAD	Melphalan	61	–	–	–	–	52	t-MDS (1 year)
PID0008	Female	Local irradiation, lenalidomide, dexamethasone	G-CSF	Melphalan	65	–	–	–	–	89	t-MDS (6 years)
PID0009	Male	Lenalidomide, bortezomib, dexamethasone	G-CSF	Melphalan	62	–	–	–	–	104	t-MDS (1 year)
PID0010	Male	Bortezomib, dexamethasone	G-CSF	Melphalan	62	–	–	–	–	88	t-MDS (3 years)

G-CSF, granulocyte colony-stimulating factor; CVAD, cyclophosphamide, vincristine, doxorubicin, dexamethasone; XRT, radiation; IFN, interferon; PBSCH, peripheral blood stem cell harvest.

PBSC samples were cultured in semiliquid methylcellulose media to generate single-cell-derived colonies. Genomic DNA from these colonies underwent WGS. We sequenced a median of 89 colonies (range = 46–128) and 38 colonies (range = 29–52) per sample for treated patients and normal donors, respectively, totaling 1,276 colonies (1,047 colonies from treated patients and 229 colonies from normal donors). WGS achieved median 29× coverage (range = 14–68×; Supplementary Fig. 1a,b). Putative somatic mutations were identified in each colony after computationally filtering potential germline variants, sequencing artifacts and mutations that are likely acquired during in vitro culture, using bioinformatic approaches modified from previous studies^5,11^. We analyzed the variant allele frequency (VAF) distribution of somatic mutations in each colony to confirm their single-cell origin. Colonies lacking a 50% VAF peak or displaying multiple peaks were excluded as likely merged colonies (Supplementary Fig. 1c). Consequently, 1,261 colonies passed the quality control and were analyzed further.

Samples from treated patients had a median of 7 driver mutations (range = 0–19) and 1 copy number aberration (CNA; range = 0–5) per sample, whereas normal controls had medians of 0 driver mutations (0–3) and 0 CNAs (0–0; Fig. 1c). The most frequently identified driver genes in treated patients were *PPM1D* and *TP53* (each in seven samples), followed by *DNMT3A* in six samples and *TET2* in five. CNAs were rare; the only recurrent abnormality was Yq deletion in three samples. In normal controls, *DNMT3A* mutations were the most frequent drivers, found in two older individuals (aged = 64 and 68 years). The notable enrichment of *PPM1D* and *TP53* mutations, both involved in the DDR, aligns with their high prevalence in therapy-related CH^18^, contrasting with the profiles in normal controls^2^.

### Mutation burden and signatures in post-treatment HSPCs

**Fig. 2:**
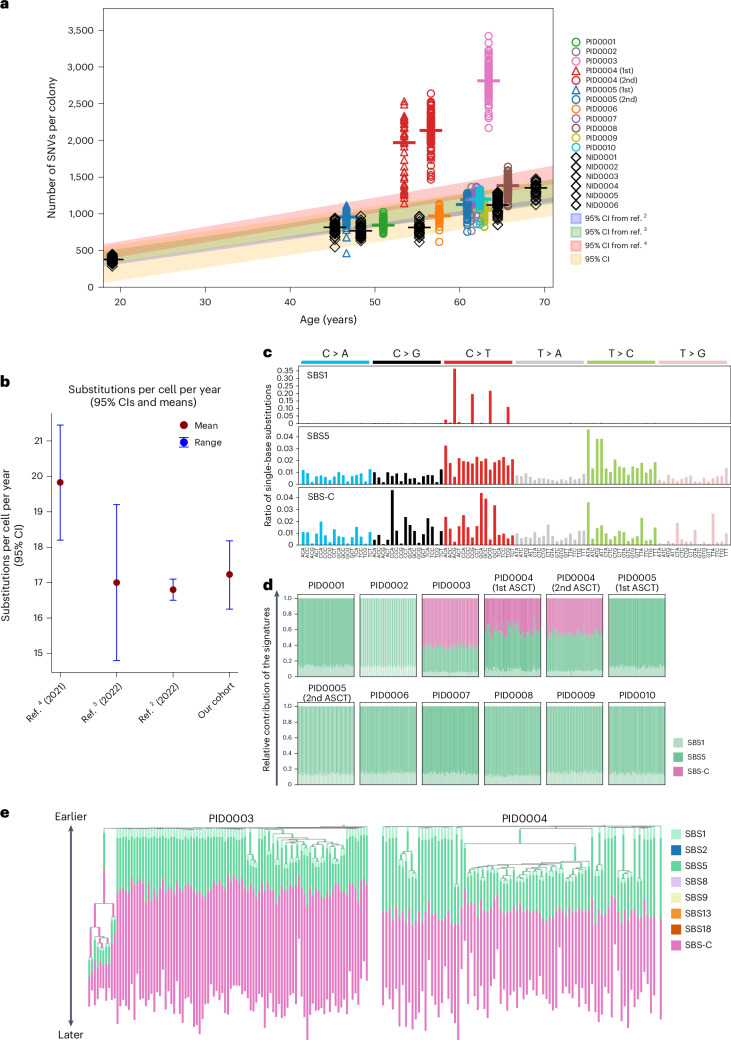
Analysis of somatic mutations and their signatures in HSPC colonies. **a**, Scatter plot of somatic SNVs per colony against age, with the red line and area representing the normal mutation rate and 95% CI from six normal donor samples. **b**, 95% CI of normal mutation rates estimated from other studies and ours. **c**, Three distinct SNV signatures deduced from the sequencing of 1,032 colonies. SBS-C is closely related, with a 90% cosine similarity, to the SBS-MM1 signature^22^. **d**, A stacked bar chart illustrating the frequency distribution of SBS1, SBS5 and SBS-C signatures across all SNVs in individual colonies. **e**, Phylogenetic trees for PID0003 and PID0004, incorporating mutation signatures. SBS-C is apparent only in mutations acquired later in the patients’ lives.

Studies have shown that the mutation rate in normal HSPCs follows a linear trajectory over time, with each single HSPC acquiring approximately 16–25 single-nucleotide variants (SNVs) per year^4,5^. Consistent with this, our normal donors’ PBSCs also showed mutation rate at 17.2 per year (95% confidence interval (CI), 16.3–18.2; Fig. 2a,b). Using these data as a benchmark, we plotted the somatic SNV counts in chemotherapy-treated HSPCs (‘post-treatment HSPCs’; Fig. 2a). Despite chemotherapy exposure, post-treatment HSPCs in eight of ten patients showed somatic SNVs matching normal age-related rates. In contrast, three samples from two patients (one sampled twice) deviated substantially, with approximately twofold or higher increases in somatic SNVs (PID0003 and PID0004; Fig. 2a). A similar trend was also observed in indels and multiple nucleotide variants (Supplementary Fig. 2). Notably, both patients had been treated with melphalan-containing therapies.

**Extended Data Fig. 2:**
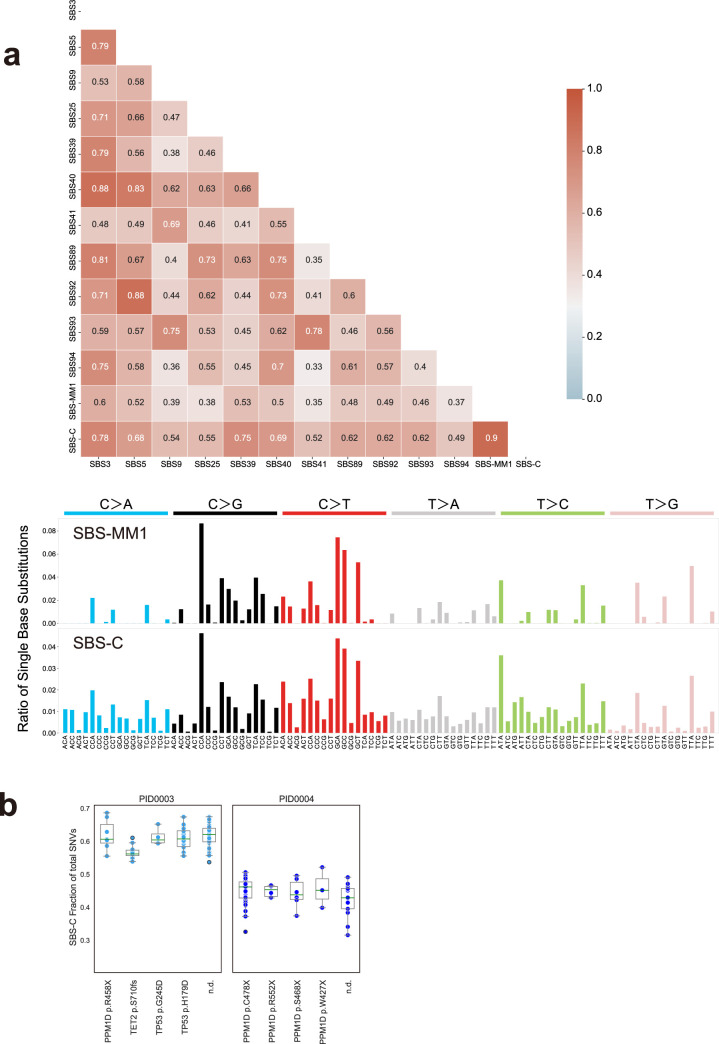
SBS-C signature profiles and driver mutation links in melphalan-treated HSPC clades. **a**, The pairwise association plot displays the cosine similarity metrics between pairs of mutation signatures, with the strength of associations represented by color intensity. The actual mutational signature plots for SBS-MM1 and SBS-C are also shown. **b**, Box plots comparing the proportion of SBS-C signatures among clades with or without driver mutations (n.d.) in PID0003 and PID0004. None of the comparisons showed statistical significance. Box plots show the median (line), upper and lower quartiles (box) ±1.5× interquartile range (whiskers). PID0003: *PPM1D* p.R458X (*n* = 7), *TET2* p.S710fs (*n* = 10), *TP53* p.G245D (*n* = 4), *TP53* p.H179D (*n* = 28), n.d. (*n* = 43), PID0004: *PPM1D* p.C478X (*n* = 40), *PPM1D* p.R552X (*n* = 6), *PPM1D* p.S468X (*n* = 6), *PPM1D* p.W427X (*n* = 3), n.d. (*n* = 16). n.d., no driver mutations.

To determine the causative relationship between melphalan treatment and the observed increase in somatic mutations, we extracted mutation signature from SNVs. In total, three mutation signatures were identified (Fig. 2c). Two were consistent with the known clock-like signatures, single-base substitution 1 (SBS1) and SBS5. SBS-C was exclusively found in the two patients exposed to melphalan. This signature showed cosine similarity of 90% with the previously identified signature SBS-MM1, which is a putative melphalan-associated signature detected in myeloma cells^22^ (Extended Data Fig. 2a). In PID0003 and PID0004, approximately 60% and 50% of mutations, respectively, were attributed to SBS-C, reflecting their elevated mutation burden compared to others (Fig. 2d). Both patients received melphalan as part of the melphalan + prednisone (MP) regimen. Notably, PID0003 had a longer treatment duration (9 months) than PID0004 (4 months), potentially contributing to the higher proportion of SBS-C mutations in PID0003’s HSPC genome. SBS-C was detected across all colonies at equal proportions, irrespective of driver mutations (Extended Data Fig. 2b), suggesting that melphalan affects HSPCs independently of their cell-cycle state, consistent with its known mechanism^23^. Phylogeny analysis shows SBS-C was acquired later in PID0003 and PID0004, reflecting post-treatment exposure at ages 53 and 52 years, respectively (Fig. 2e). These findings provide evidence of a causal link between melphalan exposure and elevated somatic mutational burden in these HSPCs. In the other patients, only clock-like signatures (SBS1 and SBS5) were observed^24^.

In contrast to melphalan, HSPCs exposed to cyclophosphamide (PID0002, PID0005 and PID0007), a similar alkylating agent, showed no increase in mutations or treatment-related signatures. This disparity likely reflects the HSC metabolism of cyclophosphamide into an inactive form via aldehyde dehydrogenase, thereby avoiding DNA damage, whereas melphalan directly forms DNA adducts in HSCs^23,25,26^. These findings underscore chemotherapy’s varied impact on the HSC genome and suggest that even agents within the same class can exert different effects on HSPC genomes in vivo.

### Clonal architecture and diversity of post-treatment HSPCs

**Fig. 3:**
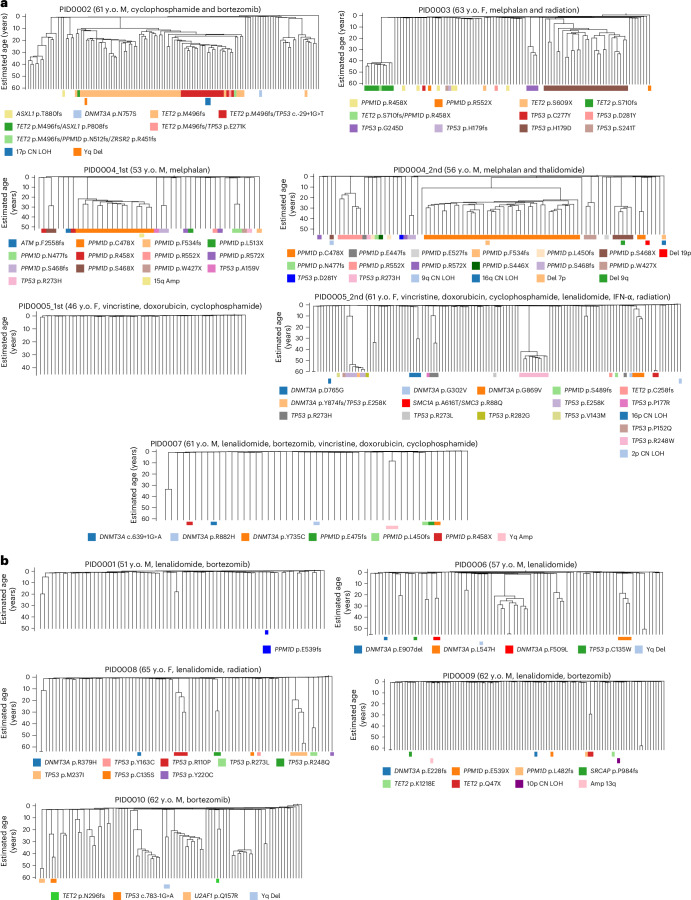
Ultrametric phylogenetic trees constructed from postchemotherapy HSPCs. These trees are based on SNVs identified in individual colonies, excluding those associated with the SBS-C signature. The trees are further detailed at the bottom, indicating the presence of driver mutations (top row) and CNAs (bottom row). **a**, Trees corresponding to samples that underwent cytotoxic chemotherapy treatments. **b**, Trees for samples treated with noncytotoxic chemotherapeutic agents. y.o., years old.

**Extended Data Fig. 3:**
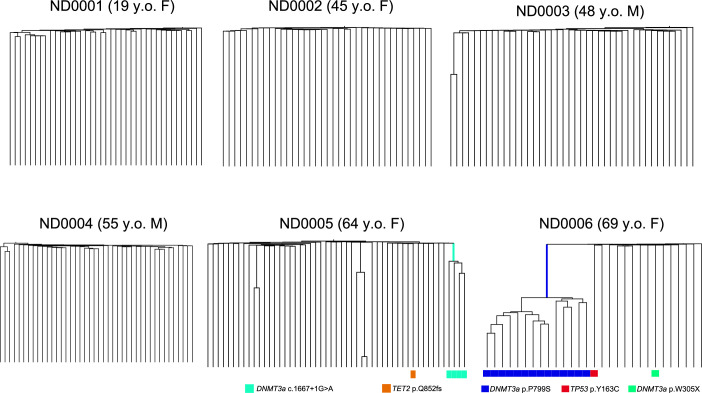
Phylogenetic trees of HSPCs from six normal donors’ PBSCs. Ultrametric phylogenetic trees of HSPCs collected from six normal donors of variable ages.

We reconstructed phylogenetic trees for post-treatment HSPCs and normal controls using shared and unique somatic SNVs from individual colonies. To avoid confounding clonal relationships and the molecular clock, we excluded treatment-related SBS-C mutations, using only clock-like signatures (SBS1 + SBS5; Fig. 3a,b and Extended Data Fig. 3). We then annotated the resulting phylogenies with known hematologic driver mutations or CNAs to clarify how expanded clades relate to these driver mutations.

**Fig. 4:**
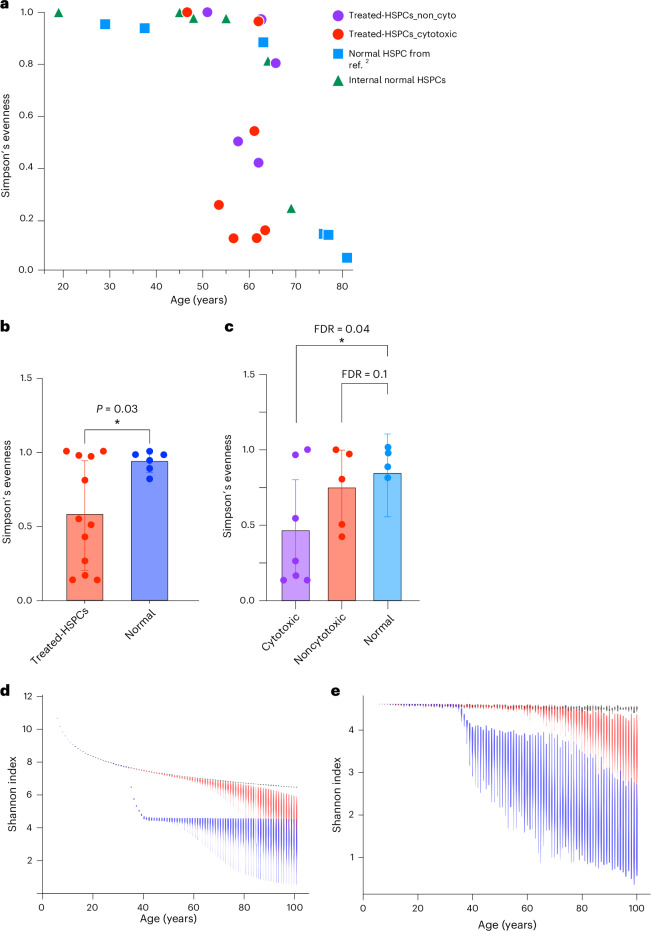
Assessment of clonal diversity in postchemotherapy HSPCs. **a**, The Simpson’s evenness index of post-treatment HSPCs from our cohort compared to indices from internal normal control and normal individuals as reported in ref. ^2^. **b**, Comparison of the Simpson’s evenness index between post-treatment HSPCs (*n* = 12) versus age-matched normal HSPCs (*n* = 6, samples from ref. ^2^ and internal normal control combined). An unpaired two-sided *t* test was performed to assess statistical significance between normal donors and treated HSPCs. Data are presented as mean ± 95% CIs, *P < 0.05. **c**, Comparison of Simpson’s evenness index between HSPCs treated with cytotoxic chemotherapy (*n* = 7), noncytotoxic chemotherapy (*n* = 5) and age-matched normal control (*n* = 4). *P* value is obtained from unpaired one-sided *t* tests. Data are presented as mean ± 95% CIs. Asterisk indicates FDR < 0.05. **d**, The simulation illustrates the projected Shannon diversity index over time for a population of 100,000 HSPCs, modeled with the Moran model. Each violin plot at each timepoint represents the results of 100 independent simulations of the model. The black line represents scenarios where acquired mutations do not affect fitness; the red line includes some mutations conferring a selective advantage; the blue line indicates the introduction of chemotherapy at approximately ages 35–40 years. **e**, The simulation displays the Shannon diversity index over time, based on a random sampling of 100 HSPCs from a pool of 100,000, considering the emergence of chemotherapy-resistant mutations (for example, *TP53* and *PPM1D*). FDR, false discovery rate.

The previous study showed that the clonal diversity of HSPCs decreases after the age of 70 years in normal individuals^2^. Some samples in the current post-treatment cohort displayed pronounced oligoclonality compared to age-matched controls, indicated by multiple large clades within the same patient (Fig. 3a,b and Extended Data Fig. 3). Quantitatively, the Simpson’s evenness index varied significantly among our patients’ samples and a subset of the samples showed a significantly lower evenness index that is comparable to that of normal individuals with age above 75 years, despite our cohort having patients aged 46–65 years (Fig. 4a,b). When we compared the evenness index between patients treated with conventional cytotoxic chemotherapies (melphalan, cyclophosphamide, doxorubicin and vincristine-treated samples) and noncytotoxic therapies (lenalidomide, thalidomide and bortezomib-treated samples), HSPCs exhibited lower evenness in patients treated with cytotoxic chemotherapies than in those receiving noncytotoxic therapies (Fig. 4c), suggesting a stronger population bottleneck of HSPCs under cytotoxic treatment.

To generalize our observation of the impact of chemotherapy on clonal diversity of HSPCs, we conducted stochastic simulation of HSPC dynamics based on the Moran model^27^. These simulations examined three scenarios—one where all acquired mutations were neutral, another with a mix of neutral and selectively advantageous driver mutations and a third including the effects of cytotoxic chemotherapy (Fig. 4d). Consistent with the prior study^2^, in the absence of chemotherapy, the natural occurrence of driver mutations contributed to a decline in diversity index around the seventh decade of life (Fig. 4d). The introduction of cytotoxic chemotherapy resulted in an immediate and sustained loss in clonal diversity (Fig. 4d). This pattern was also evident when examining the impact of sample size on the diversity index, particularly when 100 cells were randomly selected from the HSPC pool, which aligns with our experimental conditions (Supplementary Fig. 3). Furthermore, in scenarios where chemotherapy-resistant mutations emerge, such as *TP53* and *PPM1D* mutations, the reduction in clonal diversity was exacerbated, with a broader range of variation observed (Fig. 4e). This aligns with our experimental observations (Fig. 4a). Collectively, these findings indicate that cytotoxic chemotherapy accelerates the loss of clonal diversity in HSPCs and its impact persists well beyond the treatment period.

### Clone-specific analysis of mutation rate and telomeres

**Extended Data Fig. 4:**
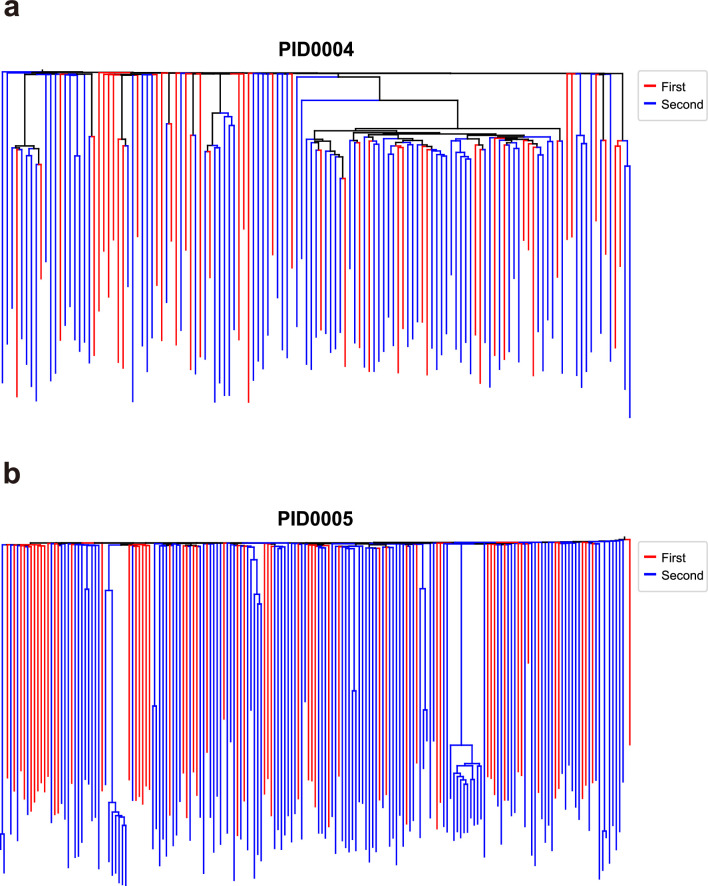
Temporal phylogenetic analysis of HSPC colonies from PID0004 and PID0005. **a**,**b**, This figure depicts the phylogenetic trees integrating colonies sampled at two different timepoints. For PID0004, the two time points are separated by an interval of 3 years (**a**), while for PID0005 the interval is 15 years (**b**). Colonies collected at the first time point are represented in red, while those from the second timepoint are denoted in blue, illustrating the clonal evolution over time.

The majority of the expanded clades in post-treatment HSPCs carried driver mutations (30 of 57 clades (53%)), contrasting previous findings in normal individuals (10 of 57 (17%; ref. ^2^); Supplementary Fig. 4). Convergent evolution of DDR pathway mutations (*TP53* and *PPM1D*) was pervasive among treated HSPCs. For instance, in PID0004, we detected six different clades evolving in parallel, each carrying different *PPM1D* mutations (Fig. 3a). This patient had a second timepoint sample taken 3 years after the first ASCT, which continued to show the convergent evolution of the same *PPM1D* mutations, indicating that all of these *PPM1D*-mutated clones engrafted after ASCT re-expanded and remobilized (Extended Data Fig. 4). Similarly, in PID0003, PID0005 and PID0008, we observed convergent evolution of multiple different *TP53*-mutated clones (Fig. 3a). These results are indicative of a strong selective pressure from chemotherapy in clonal selection of clones with DDR pathway genes.

**Extended Data Fig. 5:**
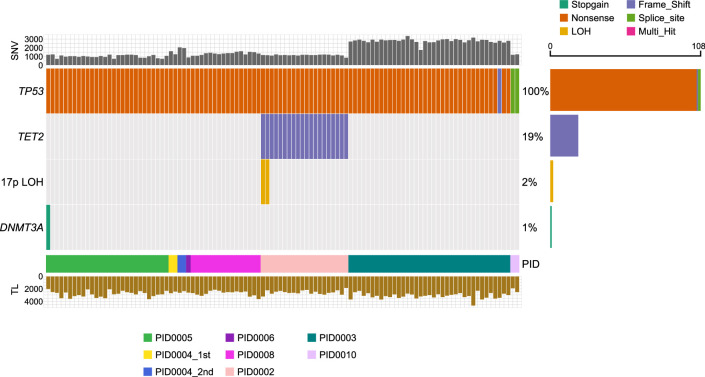
Oncoplot depicting the mutation landscape of 108 HSPC colonies harboring *TP53* mutations. Accompanying driver mutations and CNAs are also displayed. The top bar graph enumerates the total SNVs identified in each colony, while the bottom bar graph presents the estimated telomere lengths across these colonies.

In the context of myeloid malignancies, *TP53* mutations frequently co-occur with complex chromosomal aberrations. However, in the post-treatment HSPCs examined in this study, 98% of *TP53*-mutated colonies (106 of 108 colonies) showed normal copy number profiles (Extended Data Fig. 5). Only two colonies exhibited concurrent chromosomal alterations, specifically a loss of heterozygosity (LOH) on chromosome 17p (PID0002; Fig. 3a and Extended Data Fig. 5), leading to biallelic alterations in *TP53*. These data suggest that *TP53*-mutated cells do not yet display genomic instability at the CH phase and the acquisition of chromosomal aberrations emerges as late-stage leukemogenic events.

**Fig. 5:**
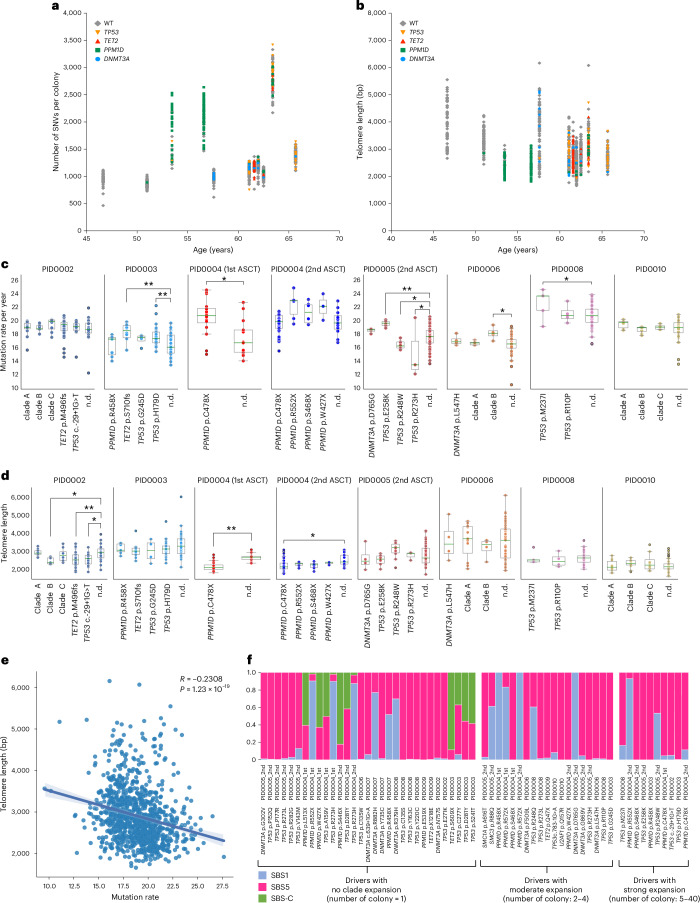
Clone-specific analysis of mutational rate and telomere length. **a**, Distribution of SNVs across individual colonies plotted against age, with colonies categorized according to the presence of driver mutations. **b**, Distribution of telomere length across individual colonies plotted against age, with colonies categorized according to the presence of driver mutations. **c**, Comparison of mutation rate (SBS1 + SBS5 counts per year) between clades with or without driver mutations and WT colonies. Statistical significance was assessed using an unpaired *t* test. Single-asterisk indicates FDR < 0.05 and double-asterisk indicates FDR < 0.01. The definition of clades without driver mutations is described in Supplementary Fig. 5. Clades with fewer than three colonies are not shown. Box plots show the median (line), upper and lower quartiles (box) ±1.5× interquartile range (whiskers). **d**, Comparison of telomere length between clades with or without driver mutations and WT colonies. Statistical significance was assessed using unpaired *t* test. Single-asterisk indicates FDR < 0.05 and double-asterisk indicates FDR < 0.01. The definition of clades without driver mutations is described in Supplementary Fig. 5. Clades with fewer than three colonies are not shown. Box plots show the median (line), upper and lower quartiles (box) ±1.5× interquartile range (whiskers). For both **c** and **d**, PID0002—clade A (*n* = 12), clade B (*n* = 7), clade C (*n* = 8), *TET2* p.M496fs (*n* = 67), *TP53* c.-29 + 1G > T (*n* = 19), n.d. (*n* = 20); PID0003—*PPM1D* p.R458X (*n* = 7), *TET2* p.S710fs (*n* = 10), *TP53* p.G245D (*n* = 4), *TP53* p.H179D (*n* = 28), n.d. (*n* = 43); PID0004 (first ASCT)—*PPM1D* p.C478X (*n* = 16), n.d. (n = 13); PID0004 (second ASCT)—*PPM1D* p.C478X (*n* = 40), *PPM1D* p.R552X (*n* = 6), *PPM1D* p.S468X (*n* = 6), *PPM1D* p.W427X (*n* = 3), n.d. (*n* = 16); PID0005 (second ASCT)—*DNMT3A* p.D765G (*n* = 4), *TP53* p.E258K (*n* = 6), *TP53* p.R248W (*n* = 10), *TP53* p.R273H (*n* = 3), n.d. (*n* = 86); PID0006—*DNMT3A* p.L547H (*n* = 4), clade A (*n* = 11), clade B (*n* = 4), n.d. (*n* = 67); PID0008—*TP53* p.M237I (*n* = 5), *TP53* p.R110P (*n* = 4), n.d. (*n* = 72); PID0010—clade A (*n* = 10), clade B (*n* = 12), clade C (*n* = 8), n.d. (*n* = 55). **e**, Scatter plot correlating mutation rate (SBS1 + SBS5 counts per year) and telomere length in each colony. Spearman correlation analysis was performed to assess the relationship between these variables. The shaded region around the regression line indicates the 95% CI for the regression estimate. **f** Assessment of the contribution of specific mutation signatures on individual driver mutations detected in treated colonies. Mutations are segregated based on the clade expansion. All SBS-C-related mutations were found in colonies with no clade expansion. n.d., colonies with no driver mutations.

To further assess the influence of driver mutations—particularly in DDR pathway genes—on genomic instability, we compared mutation rates in wild-type (WT) colonies and clades with or without driver mutations (Fig. 5a,c and Supplementary Fig. 5). Some *TP53*-mutant and *PPM1D*-mutant clades showed significantly higher mutation rates than WT colonies (Fig. 5c), although this was not universally observed across all clades with driver mutations. Additionally, certain clades without identified driver mutations also had increased mutation rates, likely due to accelerated proliferation rather than driver effects. Corroborating this hypothesis, some high-mutation clades demonstrated reduced telomere lengths (Fig. 5b,d). In addition, we observed inverse correlation between mutation rate (both overall and C > T changes at CpGs) and telomere length in HSPC colonies, further supporting the hypothesis that the increased mutation rate in some of the mutated cells is more likely attributed to accelerated clonal expansion rather than a direct result of the driver mutations per se (Fig. 5e and Supplementary Fig. 6). However, we acknowledge that these associations do not definitively establish causality, and it remains possible that increased mutation rates themselves may drive cell proliferation by acquiring mutations that confer selective advantages.

**Extended Data Fig. 6:**
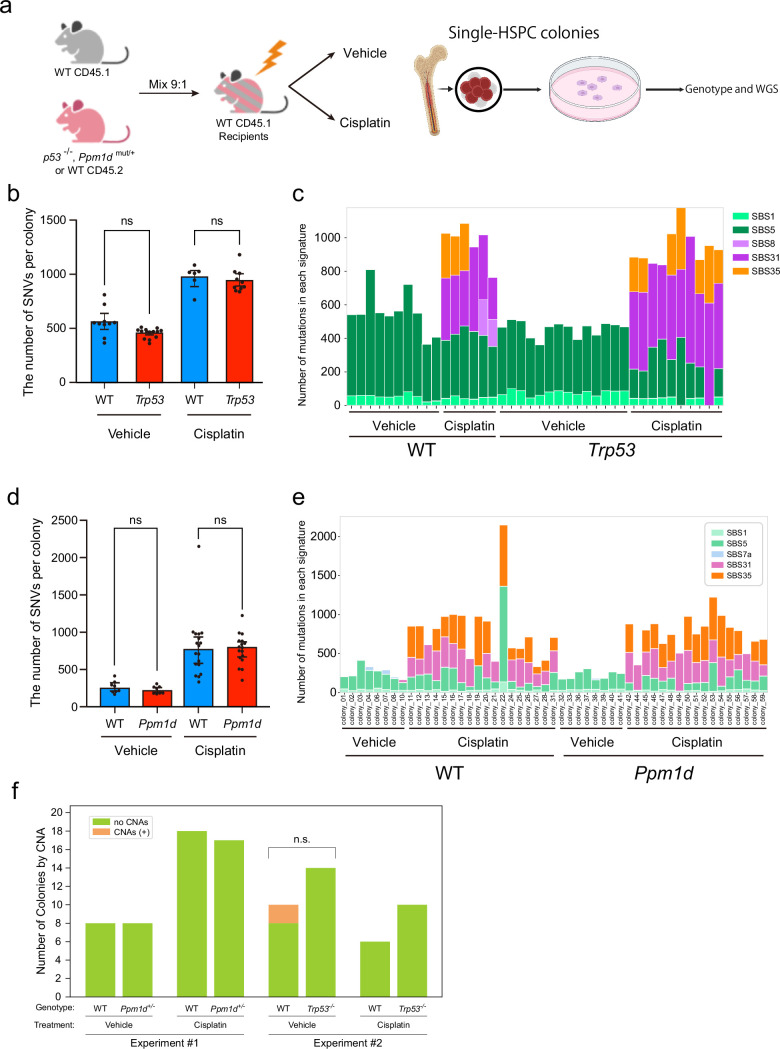
Clone-specific analysis of mutation rate and signatures using genetically engineered mouse model. **a**, The experimental schema. *Trp53* or *Ppm1d* mutant cells were transplanted with WT cells in 1:9 ratio. After the engraftment, recipient mice were treated with vehicle or cisplatin. Single-HSPC colonies were generated from bone marrow cells. Each colony was genotyped and analyzed by WGS with 30× depth to detect somatic mutations. The schematics in panel **a** are created with BioRender.com. **b**, Bar plot comparing the number of somatic SNVs between WT and *Trp53* mutant cells with or without cisplatin treatment. Statistical significance was assessed using an unpaired, two-sided *t* test. **c**, Bar plot showing the mutation signature between WT and *Trp53* mutant cells with or without cisplatin treatment. **d**, Bar plot comparing the number of somatic SNVs between WT and *Ppm1d* mutant cells with or without cisplatin treatment. Statistical significance was assessed using an unpaired, two-sided *t* test. **e**, Bar plot showing the mutation signature between WT and *Ppm1d* mutant cells with or without cisplatin treatment. **f**, Bar plot describing the numbers of colonies with or without CNAs. Statistical significance was assessed using a two-sided chi-square test. n.s., not significant.

To further validate these findings that *TP53* mutations or *PPM1D* mutations themselves are not the cause of increased mutation rate in HSPCs, we studied clone-specific mutation burden analysis using genetically engineered mouse model of therapy-related CH. Specifically, we conducted a chimeric bone marrow transplantation of WT (CD45.1) and *Trp53*^−/−^ mice (CD45.2) or WT and *Ppm1d*^mut/+^ mice (CD45.2) with 1:9 ratio. After engraftment, recipient mice were treated with either vehicle or cisplatin, and bone marrow cells were collected to generate single-HSPC colonies (Extended Data Fig. 6a). Each colony was genotyped and analyzed by WGS for clone-specific mutation rates and mutational signatures. Consistent with our human data, both *Trp53*^−/−^ and *Ppm1d*^mut/+^ cells showed mutation rates comparable to WT cells (Extended Data Fig. 6b,d). Cisplatin treatment increased mutation rates, with known platinum signatures (SBS31 and SBS35), but this increase occurred equally in both mutant and WT cells (Extended Data Fig. 6b–e). Furthermore, CNAs did not differ between mutant and WT cells, with or without cisplatin (Extended Data Fig. 6f). These findings align with our human data, indicating that at the CH stage, *TP53*-mutated and *PPM1D*-mutated cells do not inherently exhibit increased genomic instability compared to WT cells.

Next, given the evidence of chemotherapy-induced somatic mutations in HSPCs, particularly from melphalan, we investigated chemotherapy’s direct role in generating driver mutations. Using established methods, we mapped each mutation’s nucleotide context to specific signatures to estimate their contribution (Fig. 5f)^28^. Predominantly, the driver mutations corresponded with signatures SBS1 or SBS5. However, the *TET2* p.S609X mutation in PID0003 and the *PPM1D* p.S446X mutation in PID0004, identified at the second timepoint, had an 89% and 82% probability of contribution from SBS-C, respectively. Furthermore, additional driver mutations—including two *TP53* mutations in PID0003 and one *PPM1D* plus one *TP53* mutation in PID0004, detected at the first timepoint—exhibited over a 50% likelihood of association with SBS-C. Notably, the mutations linked to SBS-C did not demonstrate clonal expansion in our phylogenetic analysis (Figs. 3 and 5f). Although assigning specific mutations to chemotherapeutic agents via mutational signatures has limitations, these findings suggest that melphalan may induce some driver mutations in HSPCs, but they likely confer limited selective advantages, possibly due to their later-life induction, which limits clonal expansion.

### Mapping the clonal origin of t-MNs in PBSC samples

In the current cohort, nine of ten patients developed t-MNs with a median of 3 years (range = 1–8 years) following PBSC collection. We obtained bulk DNA from bone marrow samples at t-MN diagnosis and conducted bulk WGS (median 52× coverage). The mutational profiles and chromosomal abnormalities found in t-MN samples are detailed in Table 2.

**Table 2:** The list of driver mutations and CNAs detected in t-MN samples by bulk WGS

Sample ID	Driver 1	VAF	Shared with colonies^a^	Driver 2	VAF	Shared with colonies^a^	CNAs
PID0001	*GNAS* p.R201C	0.25	No	–	–	–	Amp 3q, Del 11q
PID0002	*TP53* c.-29 + 1G > T	0.885	Yes	*TET2* p.M496fs	0.5	Yes	Del 5q, −7, Del 17p
PID0004	*TP53* p.C135W	0.92	No	–	–	–	Del 5q, Del 6q, 17p CN-LOH
PID0005	*TP53* p.E258K	0.45	Yes	*DNMT3A* p.Y874X	0.26	Yes	Del 5q, −7, Del 17p, −18
PID0006	*TP53* p.C135W	0.833	Yes	–	–	–	Poor sample quality
PID0007	*TP53* p.V173M	0.767	No	–	–	–	Del 5q, Del 17p
PID0008	*TP53* p.M237I	0.652	Yes	*TP53* c.919 + 2T > G	0.25	Yes	Del 7q, Del 17p, −21
PID0009	*TP53* p.F134C	0.436	No	*TP53* p.G154fs	0.293	No	−3, −4, Del 19p, −21
PID0010	*WT1* p.R385fs	0.448	No	–	–	–	Normal

^a^Mutations shared with corresponding colonies are also indicated.

CN-LOH, copy-neutral LOH.

**Fig. 6:**
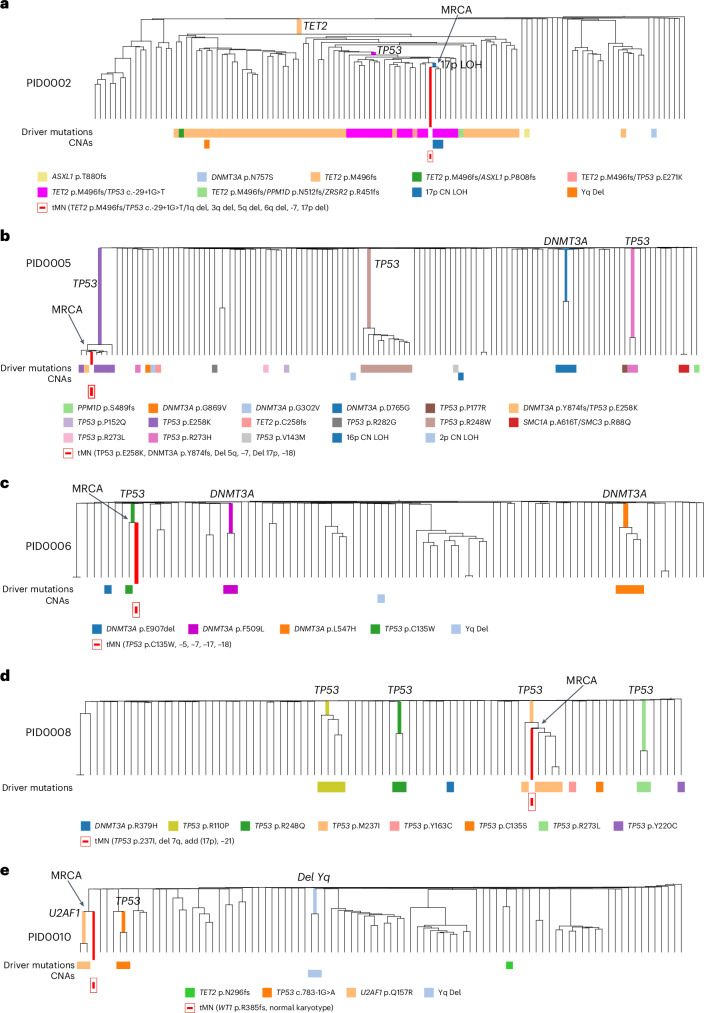
Phylogenetic relationships between post-treatment HSPCs and corresponding t-MN samples. **a**, Integrated phylogenetic tree for PID0002 highlighting the MRCA pinpointed to a clone with concurrent *TET2*, *TP53* mutations and 17p LOH. **b**, Integrated phylogenetic tree for PID0005 where MRCA was identified in *TP53* and *DNMT3A*-mutated clone. **c**, Integrated phylogenetic tree for PID0006 where the MRCA is identified within a clone possessing a *TP53* mutation. **d**, Integrated phylogenetic tree for PID0008 with the MRCA traced to a *TP53*-mutated clone. **e**, Integrated phylogenetic tree for PID0010 showing the MRCA located at a branching point preceding the acquisition of a *U2AF1* mutation.

To understand the clonal origin and evolutionary history of t-MN development, we integrated HSPC colonies and t-MN genomes for phylogenetic analysis. This approach identified the most recent common ancestor (MRCA) of t-MNs in 5 of 9 (56%) patients’ PBSC samples (PID0002, PID0005, PID0006, PID0008 and PID0010; Fig. 6 and Supplementary Fig. 7) via shared variants between colonies and t-MN genomes.

PID0002 developed therapy-related myelodysplastic syndrome (t-MDS) 1 year post-ASCT. Analysis of this patient’s HSPC colonies revealed a prominent clone with a *TET2* mutation, containing subclones with an additional *TP53* mutation. Within these subclones, two colonies also possessed 17p LOH, indicative of biallelic *TP53* alterations. The MRCA of the t-MDS clone was traced to the branchpoint of these specific clones, which carried mutations in *TET2*, *TP53* and 17p LOH (Fig. 6a and Supplementary Fig. 7a). The t-MDS sample also presented with additional CNAs such as monosomy 5 and 7, which were absent in the HSPC colonies, indicating subsequent acquisition post-MRCA.

PID0005 underwent two PBSC collections, occurring 20 and 6 years before the development of t-MDS, respectively. PBSC colonies at the first timepoint showed polyclonal HSPCs without any CH mutations. However, the second timepoint samples exhibited multiple parallel clones with *TP53* mutations and *DNMT3A* mutations. Notably, one clone with the *TP53* p.E258K mutation underwent significant expansion and included single colony with an additional *DNMT3A* p.Y874fs mutation. We traced MRCA of the t-MDS clone adjacent to the colony with this double *TP53* and *DNMT3A* mutations (Fig. 6b and Supplementary Fig. 7b). Bulk sequencing of the t-MDS sample identified both mutations with additional CNAs involving chromosomes −5, −7, −17p and −18.

PID0006’s t-MDS emerged 8 years after PBSC collection. This patient’s HSPC colonies comprised three distinct clones with different *DNMT3A* mutations and one colony with a *TP53* p.C135W mutation without any CNAs. The t-MDS sample harbored the same *TP53* mutation but also monosomy 17, indicating biallelic *TP53* alteration. Phylogenetic integration pinpointed the MRCA of the t-MDS clone with the colony harboring the *TP53* mutation (Fig. 6c and Supplementary Fig. 7c).

PID0008 presented with t-MDS 6 years postcollection. Multiple parallel *TP53*-mutated clones were identified in HSPC colonies without any concurrent CNAs. Among them, the *TP53* p.M237I mutation was also found in the t-MDS sample. The MRCA was located within the clones carrying this *TP53* mutation (Fig. 6d and Supplementary Fig. 7d). Additionally, the t-MDS sample had an essential splice site *TP53* mutation not detected in the colonies, indicating that the second *TP53* mutation was acquired post-MRCA. The shared feature of these four cases is that the sequential acquisition of secondary mutations and biallelic alteration of *TP53* is the critical factor determining malignant transformation.

The fifth case, PID0010, developed t-MDS 3 years after PBSC collection. The patient’s HSPC colonies included two with *U2AF1* mutation and two with *TP53* mutations, none of which appeared in the t-MDS sample. However, phylogenetic analysis traced the MRCA of t-MDS to a branchpoint preceding the *U2AF1* mutation, indicating the clone that led to t-MDS had diverged before acquiring *U2AF1* mutation (Fig. 6e and Supplementary Fig. 7e). Instead, the t-MDS sample contained a *WT1* mutation.

**Extended Data Fig. 7:**
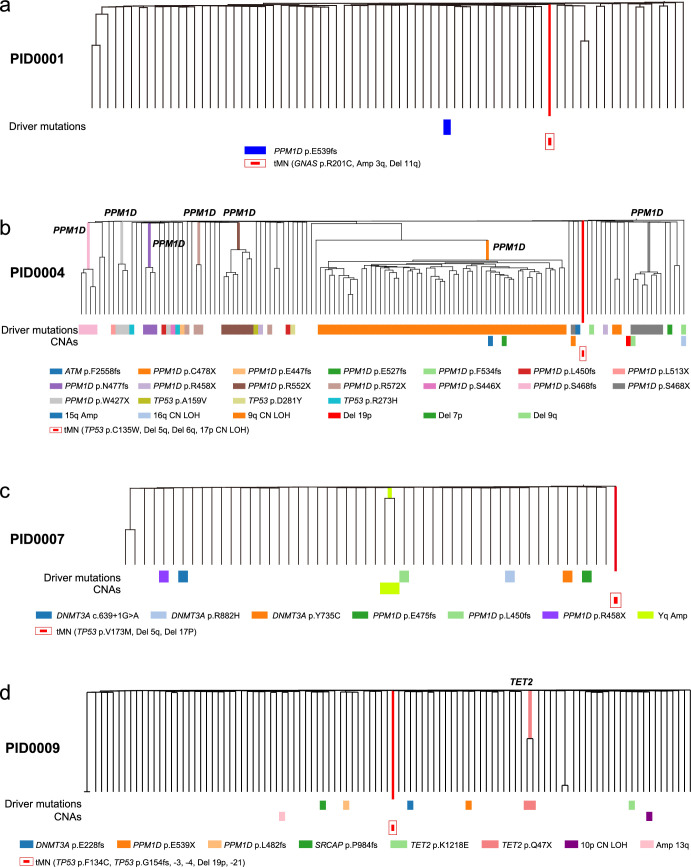
Integrated phylogenetic trees illustrating the clonal evolution of post-treatment HSPCs and corresponding t-MN samples, with a focus on cases where the most recent clonal ancestor (MRCA) could not be determined. The phylogenetic tree for PID0001 (**a**), PID0004 (**b**), PID0007 (**c**) and PID0009 (**d**), all displaying the clonal structure without a discernible MRCA.

**Extended Data Fig. 8:**
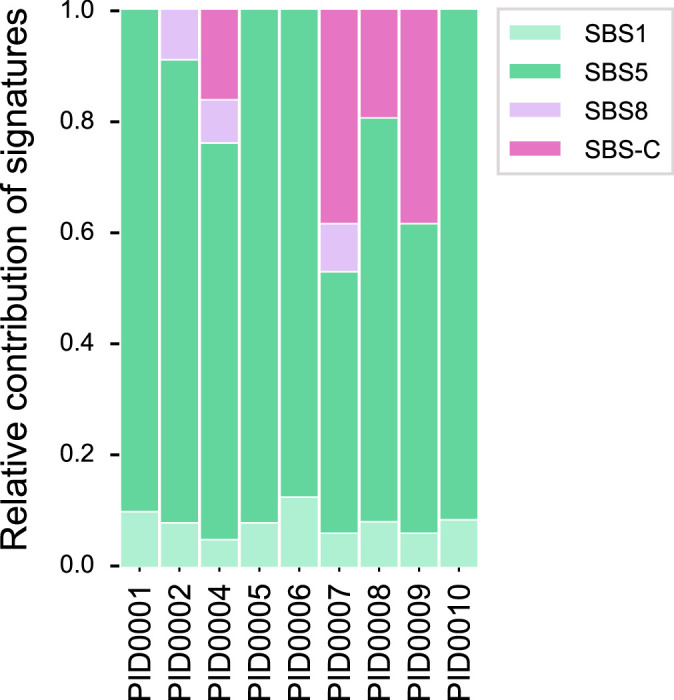
Relative contribution of the four mutational signatures in individual t-MN samples. SBS-C was detected in PID0004, PID0007, PID0008 and PID0009. PID0004 received melphalan during induction therapy. PID0008 bone marrow contained myeloma cells, therefore, SBS-C might be derived from myeloma cells treated with melphalan. For PID0007 and PID0009, the exposure to melphalan only occurred during the conditioning therapy, suggesting that t-MN might have arisen from residual bone marrow cells exposed to melphalan conditioning. PID0008 also contained SBS-C, however, the sample also contained persistent myeloma cells, making it difficult to discern whether SBS-C is from t-MN genome or myeloma cell genome.

**Extended Data Fig. 9:**
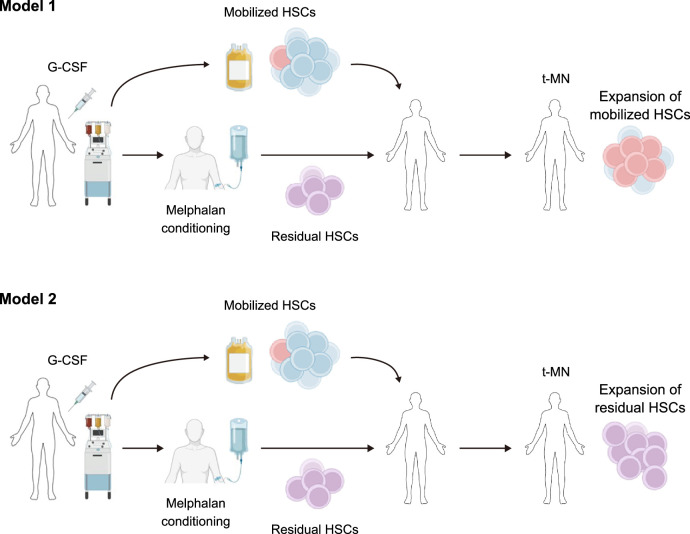
Illustration of two hypothesized models for the development of t-MNs. Model 1 depicts the scenario where t-MNs originate from HSCs that have been mobilized and subsequently transplanted. Model 2 represents the alternative pathway where t-MNs develop from residual HSCs remaining in the bone marrow, which were not mobilized and thus are subject to high-dose melphalan conditioning regimens. These schematics are created with BioRender.com.

In the other four cases (PID0001, PID0004, PID0007 and PID0009), phylogenetic analysis did not identify an MRCA in the studied colonies (Extended Data Fig. 7 and Supplementary Fig. 8). These t-MN clones diverged early and shared few mutations with HSPC colonies, an unexpected finding given that some colonies carried high-risk CH mutations (*TP53*, *PPM1D*). Given the limited number of analyzed colonies (~100 per patient), these results may reflect sampling bias. Alternatively, t-MNs may arise from nonmobilized bone marrow HSPCs. We examined t-MN mutation signatures under the hypothesis that residual HSPCs would show a melphalan-induced footprint from conditioning regimen. Indeed, approximately 40% of PID0007’s and PID0009’s t-MN mutations were SBS-C, indicative of melphalan exposure (Extended Data Fig. 8). Given that PID0007 and PID0009 did not receive melphalan during the induction phase, it suggests that the t-MN originated from bone marrow HSPCs that were subjected to high-dose melphalan during the conditioning regimen. Although these findings require validation in a larger cohort, they suggest the following two distinct developmental pathways for t-MNs post-ASCT: while most arise from clones within PBSCs, a minority may develop from nonmobilized bone marrow HSPCs that underwent conditioning therapy (Extended Data Fig. 9).

## Discussion

The clinical toxicity of cancer chemotherapy can manifest in the hematopoietic system, ranging from transient myelosuppression to the devastating development of t-MNs. We utilized a single-colony WGS approach to evaluate the influence of cancer chemotherapy on the genome and clonal dynamics of human HSPCs. Although the chemotherapies examined here are not exhaustive, our analysis identified two significant effects on normal HSPCs: direct mutagenesis and clonal architecture remodeling via the selective expansion of DDR-mutated CH.

For direct mutagenesis, melphalan treatment was associated with the induction of treatment-related mutations in HSPC genomes, potentially including driver mutations. No significant mutagenic effects were observed for other chemotherapies tested in this study, although it remains possible that the signature analysis may have missed detecting treatment-induced mutations if their proportion was small. Despite also being an alkylating agent, cyclophosphamide did not elicit treatment-induced mutations, likely because HSCs metabolize it into an inactive form via aldehyde dehydrogenase^25^. These observations align with the established clinical practice of administering cyclophosphamide three days after allogeneic HSC infusion (the post-Cy regimen)^29^, thereby preventing graft-versus-host disease without compromising engraftment. Furthermore, its use in breast cancer correlates with a markedly lower incidence of t-MNs compared to melphalan^30^.

The second effect, clonal remodeling of HSPCs, was observed more broadly with other cytotoxic chemotherapies, even without substantial mutagenic effects. Compared to previously published data on healthy, untreated individuals^2^, treated HSPCs—particularly those exposed to cytotoxic agents—showed marked loss of clonal diversity, driven by the expansion of multiple clones harboring driver mutations such as *TP53* and *PPM1D*. This observation aligns with prior studies demonstrating chemotherapy’s role in promoting the clonal selection of *TP53* and *PPM1D* mutated HSCs^19–21^. The clonal heterogeneity of chemotherapy-treated HSPCs in patients aged 40–50 years resembled that of healthy individuals over 70 years, suggesting chemotherapy might accelerate the clonal aging of HSCs. Mathematical simulations of HSC dynamics, with and without chemotherapy, corroborated this finding, reinforcing the notion that chemotherapy reduces HSC diversity. Thus, chemotherapy’s temporal repercussions extend beyond immediate cytotoxic effects, implying a long-term reshaping of HSC architecture that parallels the natural genetic evolution of aging.

**Extended Data Fig. 10:**
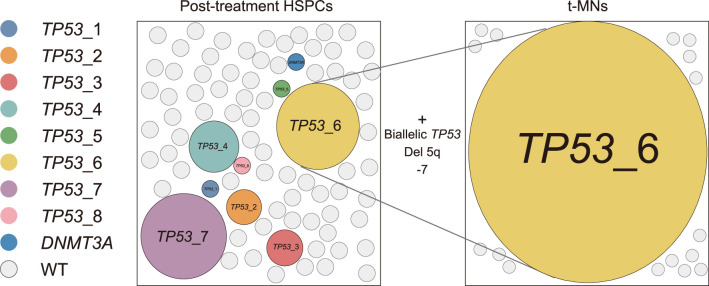
Clonal evolution of t-MNs. Illustrative representation of clonal evolution from postchemotherapy HSPCs to t-MNs (PID0008). The figure portrays the parallel evolution of multiple HSPC clones, each harboring different TP53 and DNMT3A driver mutations of varying clonal sizes, interspersed with wild-type (WT) cells. It highlights one clone that develops biallelic TP53 alterations and acquires additional copy number alterations, ultimately undergoing selective clonal expansion with the transformation to t-MNs.

One such long-term consequence of chemotherapy is the development of t-MNs. Among the chemotherapy’s two observed effects, clonal remodeling clearly impacts t-MN development. We found that chemotherapy-treated HSPCs frequently undergo branching convergent evolution, resulting in the emergence of multiple parallel clones with *TP53* mutations, each carrying potential leukemic risk (Extended Data Fig. 10)^31,32^. The comparative genomic analysis of matched t-MNs and treated HSPCs revealed that the clonal origin of t-MNs can be traced back to a single-HSPC clone with *TP53* mutation. Thus, chemotherapy reshapes the clonal architecture, inducing multiple parallel high-risk clones and allowing a single clone’s selective expansion into t-MNs. This raises crucial questions about the genetic or epigenetic factors underlying such selective dominance. Our data suggest that clones acquiring biallelic *TP53* alterations (through a second mutation or LOH) and additional chromosomal changes possess the highest leukemic potential, consistent with the known high prevalence and poor prognosis of biallelic *TP53* alterations in t-MNs^21,33^. These findings underscore the critical role of secondary mutations in clonal selection and malignant transformation in t-MNs (Extended Data Fig. 10). Clinically, they highlight the importance of monitoring not only *TP53*-mutated clonal proliferation via bulk sequencing but also allelic status, which likely requires single-cell genotyping. This approach supports early identification of clones poised to evolve into t-MNs. Furthermore, most *TP53*-mutated cells showed no genomic instability during the CH stage, suggesting that targeting *TP53* mutations at this phase may be more effective than intervening at the t-MN stage, after additional chromosomal and genetic aberrations accumulate. Consequently, early interception during the CH stage—rather than waiting for the malignant transformation of these high-risk *TP53* clones—could improve patient outcomes.

Direct mutagenic effects in t-MN pathogenesis remain unclear. Although melphalan-induced mutations may include oncogenic drivers, many are likely random passenger mutations. Additionally, our phylogenetic analysis suggests that treatment-induced driver mutations may lack a strong selective advantage, possibly due to their late-life acquisition. Further systematic studies are needed to elucidate the contribution of chemotherapy-induced direct mutagenesis in t-MN development.

In the majority of cases, our integrated phylogenetic analysis traced t-MN clonal origins to PBSCs. However, in a subset of cases, clonal origins remained undetermined; notably, two t-MN samples of those cases showed a melphalan signature, suggesting they arose from nonmobilized bone marrow HSPCs, consistent with the findings in ref. ^34^. We thus propose two developmental pathways for t-MNs following ASCT—one originating from transplanted PBSCs and another from nonmobilized bone marrow HSPCs. Clinically, this implies that screening PBSC samples for high-risk CH mutations may not always predict the emergence of t-MNs.

This study has several limitations. First, it only included patients with MM, restricting chemotherapies to those used in MM treatment. A broader analysis is needed to fully characterize the mutagenic effects of a wider range of cancer chemotherapies. Second, the study may be underpowered to assess how factors like germline mutations, drug metabolism and chemotherapy dosing affect HSPC genomic integrity and clonal dynamics. Similarly, mutation signature analysis may overlook subtle changes from hidden exposures. Third, we did not analyze samples from patients who did not develop t-MNs. Comparing clonal diversity in PBSCs from patients who did and did not develop t-MNs could yield further insights into t-MN risk. Although bulk sequencing of PBSCs from ASCT patients has been examined^35^, it does not fully capture HSPC clonal diversity, underscoring the need for single-cell approaches. Lastly, single-cell colony sequencing may have introduced sampling bias. Future studies using direct single-cell sequencing without colony formation could provide an unbiased assessment of HSPC clonality.

In summary, our study revealed differential effects of cancer chemotherapy on the HSPC genome, underscoring the need for further systematic research evaluating chemotherapy’s impact on the HSC genome and its long-term consequences. We also found that chemotherapy accelerates the loss of clonal diversity in HSPCs by promoting the expansion of multiple clones harboring DDR pathway mutations, notably *TP53* and *PPM1D*. Among these, one clone eventually transforms into t-MNs. Further investigation is warranted to identify the precise genetic or epigenetic determinants dictating this transformation and clonal competition. Additionally, establishing clinical strategies to monitor patients exposed to high-risk chemotherapy is crucial for the early detection and potential interception of emergent t-MNs.

## Methods

### Patient samples

Aliquots of cryopreserved PBSCs from patients with MM who underwent ASCT were utilized to culture single-HSPC colonies. For those patients who subsequently developed t-MNs, genomic DNA was extracted from their bone marrow mononuclear cells for WGS analysis. For normal samples, cryopreserved PBSCs from healthy donors who donated for allogeneic SCT were used. Written informed consent for the collection and analysis of samples was obtained from all participating patients. The study protocols were conducted in accordance with ethical guidelines and received approval from The University of Texas MD Anderson Cancer Center’s institutional review board (PA15-0400).

### Single-HSPC colony formation and DNA extraction

Cryopreserved PBSCs were carefully thawed and then cultured at 37 °C using MethoCult H4435 Enriched Medium (STEMCELL Technologies), adhering to the manufacturer’s instructions. After a period of 14–18 days of incubation, colonies were identified using microscope, and individual single-cell-derived colonies were collected and suspended in PBS. Genomic DNA was then extracted from these colonies using the DNeasy Blood & Tissue Kit (Qiagen, 69506), following the protocol provided by the manufacturer. The quality of the extracted DNA was assessed using Qubit fluorometric quantitation and/or TapeStation analysis before WGS.

### WGS and read alignment

WGS was performed in the Advanced Technology Genomics Core Facility at MD Anderson Cancer Center. Briefly, Illumina-compatible, uniquely dual-indexed libraries were prepared from 200 ng of RNase-treated DNA. The DNA was sheared to approximately 350 bp using Diagenode Bioruptor Pico, and then, libraries were prepared using the KAPA hyper library preparation kit (Roche Sequencing Solutions). The libraries were amplified with three to eight cycles of PCR, then assessed for size distribution using the 4200 TapeStation High Sensitivity D1000 ScreenTape (Agilent Technologies) and quantified using the Qubit dsDNA HS Assay Kit (Thermo Fisher Scientific). Equimolar quantities of the indexed libraries were multiplexed, 31–32 libraries per pool. The pool was then quantified by qPCR using the KAPA Library Quantification Kit (KAPA Biosystems) and sequenced on the Illumina NovaSeq 6000 S4-300 flow cell using the 150 nt paired-end format. WGS with low input material was carried out using Illumina NovaSeq with 150 bp paired-end sequencing, which provided approximately 28× coverage per colony sample. Quality control for the raw sequence data, including trimming of adapters, was conducted using Trim Galore, version 0.6.5. The sequencing reads were then aligned to the human reference genome (NCBI Build 37) using the BWA-MEM algorithm. Sequence deduplication was carried out employing the Picard MarkDuplicates function.

### Somatic mutation calling

Variant calling was conducted using the Genome Analysis Toolkit (GATK) Mutect2 (ver 4.2.0.0) against a pool of unmatched normal control samples^36^. After Mutect2 filtration, additional custom filters were applied to refine mutation calls, aiming to eliminate germline variants, sequencing errors, artifacts, misalignments and mutations acquired during the culture. To filter germline variants, we adopted the methodology used in ref. ^11^. Briefly, we fitted a binomial distribution model to Mutect2-filtered variants and total depth across all samples from a single patient. A one-sided exact binomial test was performed with the null hypothesis that the variants would display a binomial distribution with a probability of 0.5 (0.95 for sex chromosomes in males). Variants with *P* values greater than 10^−10^ were classified as germline. Additional variant filtering was conducted following the method described in ref. ^5^, and consists of (1) removal of mutations located within ten base pairs of each other, (2) mutations exhibiting sequencing coverage of fewer than five reads for autosomes or for X chromosomes in females and fewer than three reads for sex chromosomes in males were assigned a status of ‘NA’ in the respective sample. To minimize the risk of sequencing artifacts, any mutations bearing this ‘NA’ status identified in more than five samples within a single patient were excluded from the dataset, (3) removal of mutations with a mean VAF across all samples containing mutant reads of 0.3 or less, and (4) removal of variants with a VAF below 0.1 observed in over 10% of samples.

Driver mutations for CH were identified from a prespecified list of hematopoietic driver mutations previously defined in ref. ^37^. Additionally, we screened for mutations that were recently identified as positively selected genes from ref. ^38^.

### Copy number analysis

Copy number profiles were generated from WGS data using the AscatNGS pipeline (https://github.com/cancerit/ascatNgs). One of the colonies that belonged to the unexpanded clades from the same patients was used as a matched normal. These regions that have larger than 5 Mbp in this process are considered true CNAs. For mouse colony copy number analysis, due to the usage of inbreeding mouse cohort, B-allele frequency or LOH analysis was impractical in our experiment. Our CNV analysis mostly focused on amplification or deletion events in autosomal chromosomes, utilizing the FACETS method. All samples underwent analysis for copy number variations three to five times, using different matched control samples from vehicle-treated groups with identical genetic backgrounds. To identify definitive CNA regions, we focused on areas consistently seen in over 50% of the trials. CNA areas exceeding 3.0 × 10^6^ base pairs in a single region and total above 5.0 × 10^6^ base pairs per chromosome were subsequently validated through direct observation.

### Estimation of telomere length from WGS data

Based on the WGS data from each colony, telomere length was estimated using the Telomerecat algorithm^39^.

### Mutation signature analysis

We applied SigProfiler to our WGS data^40^ and cross-referenced the extracted signatures with the known COSMIC signatures (https://cancer.sanger.ac.uk/cosmic/signatures/SBS/). Mutation signatures that show cosine similarity of 0.80 or lower were considered as new signatures. Subsequently, both new and previously identified signatures were reassigned to the samples, with careful consideration of each patient’s clinical history. To confirm the presence of each mutated signature in the samples and estimate its contribution, fitting algorithms from SigProfilerExtractor^41^ and mmSig^42^ were utilized, which showed consistent results.

### Phylogenetic tree reconstruction

Somatic mutations in all samples were described in the PHYLIP format files, and the alleles on the genomic positions where the variants were not called were considered to be reference alleles. The putative allele sequences of the hypothetical fertilized zygote, which consist of reference alleles after removal of the germline mutations, were also added as an outgroup of the samples. The phylogenetic trees for these samples are inferred as follows.

PHYML (version 3.3.20211231) was used to infer the cladogram based on the maximum-likelihood method. The number of bootstraps to estimate the initial cladogram for the samples was 100. The node between the outgroup and other samples is set as root. After the CNV analysis, PHYLIP files for each clade on the initial phylogenetic tree were created, and the alleles that existed on the LOH region of more than a single sample were removed. Then, the cladogram for the clade was re-evaluated with PHYML with a bootstrap number of 1,000. These reevaluations of cladogram were performed for each clade in descending order.

### Branch length adjustment on phylogenetic tree

Based on the cladogram, ancestral sequence reconstruction was performed for all ancestry nodes with the phangorn library in the R language. Mutational signature analysis was conducted on the somatic mutations identified in colony samples and the inferred ancestral sequences. This analysis aimed to assess the relative impacts of intrinsic and extrinsic factors on each node, quantifying their respective influences. Then, we evaluated the differences in the signature counts between each pair of a child node and a parent node by subtracting the signature counts on the parent node from the child node. If a negative value was computed for any signature count on the branch, two criteria were independently assessed for each branch—(1) whether the value fell within the range of −1 to 0 or (2) whether the absolute value of the negative value was less than 5% of the total signature count for the branch.

When either condition was met, the negative value was regarded as an artifact. All the negative values on each branch in our phylogram met either the first or second criterion, and these negative counts were consequently treated as zero.

After this process, the sum of the counts of SBS1 and SBS5, both clock-like signatures, is applied to the branch length to represent the evolutionary time for these phylograms.

### Simulating HSPC population dynamics and clonal diversity

We adopted the Moran model to represent a turnover of a healthy HSPC population over age with the accumulation of mutations, where a cell for death is randomly chosen and another cell is randomly chosen for division according to the fitness proportionate selection during one time step^27^. This model keeps the number of the HSPC population constant. Let us begin with *N* active HSPCs after tissue maturation and assume that one time step corresponds to 1/*n* year. Then, each HSPC divides once per year on average. We assumed that by age 5, most HSPC clones are already established and continue to constitute the majority of the HSPC pool throughout the individual’s lifetime^5^, and each cell is defined as an independent clone in the initial population. As time passes, some clones may expand in the HSPC population and others may go extinct, leading to the reduction in clonal diversity. Initially, all HSPCs have the same fitness, 1.0, but their fitness additively increases with *s* when a driver mutation occurs during a cell division, where the mutation rate per division is denoted by *u*. Therefore, the probability that the *k*th cell with *i*_*k*_ driver mutations is chosen for division is given by

$$\Pr \left({\rm{division}}\right)=\frac{1+{i}_{k}s}{\Gamma },$$

where $\Gamma =\sum _{j}(1+{i}_{j}s)$. When *s* = 0, the probability of division is the same among all cells irrespective of their mutations, indicating that the population dynamics is neutral. Note that the probability that a cell is chosen for death is the same among all HSPCs in this model.

Next, we introduced the effect of chemotherapy in HSPC population. The chemotherapy damages HSPC population to decrease the total population number into $\varepsilon n\left(\varepsilon < 1\right)$ during the chemotherapy. After the treatment, the total number is assumed to be recovered within *β* years. We consider the following two different scenarios for the chemotherapy: (1) the probability that a cell is chosen for death is the same among all HSPCs as in the healthy state and (2) cells that have a specific mutation are resistant to death during the chemotherapy.

In the latter case, the mutation resistant to death by the chemotherapy would emerge before or during the chemotherapy with probability *m* per division. The probability that the *k*th cell is chosen for death during the chemotherapy is given by ${d}_{k}/{\sum }_{j}{d}_{j}$, where *d*_*k*_ is *w* (*w* < 1) for cells that have the resistant mutation, and 1.0 for cells that do not have the resistant mutation.

### Mouse bone marrow transplantation and single-colony WGS

The study protocol involving mouse models was approved by the Institutional Animal Care and Use Committee of our institution. The development of *Ppm1d*^R451X/+^ mice and *Trp53*^−/−^ mice was previously reported^19^. For the bone marrow transplantations, donor bone marrow from age and sex-matched 5-to-10-week-old *Ppm1d*^R451X/+^ (CD45.2), *Trp53*^*−/−*^ (CD45.2) or wild-type control littermates were mixed with bone marrow from wild-type mice (CD45.1) in a 10:90 ratio, with a total of 3 × 10^6^ cells transplanted into each recipient mouse. Mixed bone marrow cells were retro-orbitally injected into 5-week-old to 10-week-old lethally irradiated CD45.1 recipient mice (split-dose of 1,100 cGy total, separated by 4 h). After 6 weeks, engraftment was confirmed by analyzing the peripheral blood for the baseline chimerism. Then, recipient mice were randomly treated with either cisplatin (4 mg kg^−1^, intraperitoneal) or vehicle once a week starting at 6 weeks post-transplant, for five consecutive weeks. At 11 weeks post-transplant, recipient mice were sacrificed to collect bone marrow cells by crushing the long bones (tibias and femurs) with a mortar and pestle in Hank’s buffered salt solution. Lineage-positive cells were depleted by bead purification using the BD Pharmingen Biotin Mouse Lineage Panel. A total of 1,000 lineage-negative bone marrow cells were seeded per well in a 6-well plate in 1.5 ml methylcellulose media (GF M3434) as described above. After 14 days of culture, colonies were collected and DNA was extracted using the Genomic DNA Clean & Concentrator (Zymo, D4010). Each colony was genotyped using PCR assay for *Ppm1d* and *Trp53* status using the primer sequences below (Supplementary Table 1). Mouse DNA was then subjected to WGS with an average depth of 30×.

### Statistics and reproducibility

Error bars in the figures represent s.d. unless otherwise specified. Differences between the two datasets were assessed using a two-sided unpaired *t* test or a Mann–Whitney test for non-normally distributed data. Statistical significance was defined as *P* < 0.05. No statistical methods were used to predetermine sample sizes. During WGS data analysis, colonies lacking symmetrical VAF histograms were excluded from subsequent data processing. The experiments were not randomized, and investigators were not blinded to allocation during experiments or outcome assessment. Data collection and analysis were conducted without blinding to experimental conditions.

### Reporting summary

Further information on research design is available in the Nature Portfolio Reporting Summary linked to this article.

## Online content

Any methods, additional references, Nature Portfolio reporting summaries, source data, extended data, supplementary information, acknowledgements, peer review information; details of author contributions and competing interests; and statements of data and code availability are available at 10.1038/s41588-025-02235-w.

## Supplementary information

Supplementary Information Supplementary Figs. 1–8.

Reporting Summary

Supplementary Table 1 Primer sequences utilized for genotyping transgenic mice.

## Data Availability

WGS data from all colonies are available at the Sequence Read Archive (SRA) with the project numbers PRJNA1058953 and PRJNA1206464.

## Code availability

The computational methods, procedures, and analyses described above have been implemented using custom R, Python, and Bash scripts. These scripts are publicly accessible on GitHub (https://github.com/Huryu-MDA/ClonalEvol_after_chemotherapy (ref. ^43^) and https://github.com/KoichiSaeki/uryu-et-al-2025-moranmodel (ref. ^44^)).
