# Author Correction: Clonal evolution of hematopoietic stem cells after autologous stem cell transplantation

**DOI:** 10.1038/s41588-025-02328-6

**Published:** 2025-08-11

**Authors:** Hidetaka Uryu, Koichi Saeki, Hiroshi Haeno, Chiraag Deepak Kapadia, Ken Furudate, Jyoti Nangalia, Michael Spencer Chapman, Linda Zhang, Jennifer Padilla, Li Zhao, Joanne I. Hsu, Chong Zhao, Shujuan Chen, Tomoyuki Tanaka, Zongrui Li, Satoko Ogata, Sarah Hanache, Hui Yang, Courtney DiNardo, Naval Daver, Naveen Pemmaraju, Nitin Jain, Farhad Ravandi, Jianhua Zhang, Xingzhi Song, Erika Thompson, Hongli Tang, Latasha Little, Curtis Gumbs, Robert Z. Orlowski, Muzaffar Qazilbash, Kapil Bhalla, Simona Colla, Hagop Kantarjian, Rashmi Kanagal-Shamanna, Carlos Bueso-Ramos, Daisuke Nakada, Gheath Al-Atrash, Jeffery Molldrem, P. Andrew Futreal, Elizabeth Shpall, Margaret Goodell, Guillermo Garcia-Manero, Koichi Takahashi

**Affiliations:** 1 Department of Leukemia, The University of Texas MD Anderson Cancer Center, Houston, TX USA; 2 Tokyo University of Science, Division of Integrated Research, Research Institute for Biomedical Sciences, Noda, Japan; 3 Department of Molecular and Cellular Biology, Baylor College of Medicine, Houston, TX USA; 4 Wellcome Sanger Institute, Wellcome Genome Campus, Hinxton, UK; 5 Barts Cancer Institute, Queen Mary University of London, London, UK; 6 Department of Genomic Medicine, The University of Texas MD Anderson Cancer Center, Houston, TX USA; 7 Department of Medicine, Dana–Farber Cancer Institute, Harvard Medical School, Boston, MA USA; 8 Department of Hematopathology, The University of Texas MD Anderson Cancer Center, Houston, TX USA; 9 Department of Genetics, The University of Texas MD Anderson Cancer Center, Houston, TX USA; 10 Department of Lymphoma and Myeloma, The University of Texas MD Anderson Cancer Center, Houston, TX USA; 11 Department of Experimental Therapeutics, The University of Texas MD Anderson Cancer Center, Houston, TX USA; 12 Department of Stem Cell Transplantation and Cellular Therapy, The University of Texas MD Anderson Cancer Center, Houston, TX USA; 13 Department of Molecular and Human Genetics, Baylor College of Medicine, Houston, TX USA

**Keywords:** Genetics research, Acute myeloid leukaemia

Correction to: *Nature Genetics* 10.1038/s41588-025-02235-w, published online 1 July 2025.

In the version of this article originally published, Linda Zhang and Jennifer Padilla (both at the Department of Molecular and Cellular Biology, Baylor College of Medicine, Houston, TX, USA) did not appear in the author list or author contributions. The error has been corrected in the HTML and PDF versions of the article.

