# Supplementary material for: Clonal evolution of hematopoietic stem cells after autologous stem cell transplantation
Source: Nat Genet. 2025 Jul 1;57(7):1695–707. doi: 10.1038/s41588-025-02235-w (PMC12283406; doi:10.1038/s41588-025-02235-w)
Supplement: Supplementary file 1 — Supplementary Figs. 1–8. [file 41588_2025_2235_MOESM1_ESM.pdf]

nature genetics

Supplementary information

https://doi.org/10.1038/s41588-025-02235-w

Clonal evolution of hematopoietic stem cells after autologous stem cell transplantation

In the format provided by the authors and unedited

Table of Contents

Supplementary Figure 1. Coverage and VAF Histogram Validation of Single-Cell Colonies 3

Supplementary Figure 2. Age-related distribution of somatic indels and MNVs in HSPC colonies........................ 5

Supplementary Figure 3. Simulation of Shannon diversity index over time. 6

Supplementary Figure 4. Comparative analysis of driver-mutant clades in normal and treated HSPCs................ 7

Supplementary Figure 5. The definition of clades without obvious driver mutations. 8

Supplementary Figure 6. Scatter plot correlating telomere length and C>T counts. 9

Supplementary Figure 7. Phylogenetic trees of HSPC colonies and matched t-MN genomes with identified MRCA.. 14

Supplementary Figure 8. Phylogenetic trees of HSPC colonies and corresponding t-MN genomes lacking a distinct MRCA.. 18

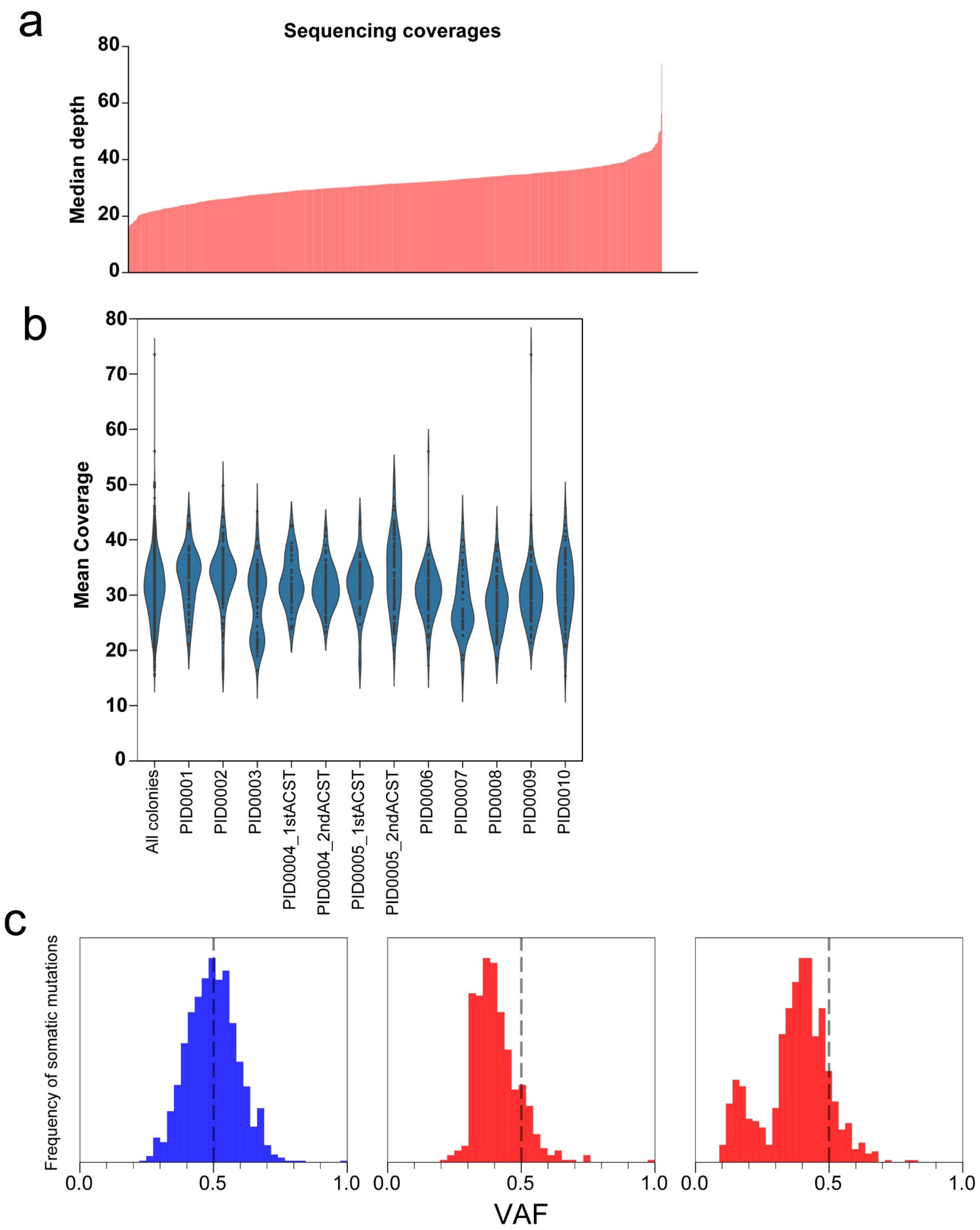

a
Sequencing coverages
Median depth
0
20
40
60
80
b
Mean Coverage
0
10
20
30
40
50
60
70
80
All colonies
PID0001
PID0002
PID0003
PID0004_1stACST
PID0004_2ndACST
PID0005_1stACST
PID0005_2ndACST
PID0006
PID0007
PID0008
PID0009
PID0010
c
Frequency of somatic mutations
0.0
0.5
1.0
VAF

Supplementary Figure 1. Sequencing Coverage Across All Samples and Validation of Single-Cell-Derived Colonies Using VAF Histograms.
(**a**) Bar graph illustrating the median sequencing coverage of mapped regions across all 1,032 colonies. This data demonstrates consistent coverage across the dataset, ensuring that the melphalan-treated colonies were not outliers due to insufficient sequencing depth. (**b**) Violin plot showing the sequencing coverage based on the patient sample. The sequencing coverage was comparable among all patients. (**c**) Analysis of variant allele frequency (VAF) for somatic mutations in HSPC colonies. Representative histograms of VAF of somatic SNVs detected in single-cell colonies. The left histogram represents the sample that shows a VAF peak at 50%, indicating a single-cell-derived colony. The middle histogram shows a VAF peak lower than 50%. The right histogram showed two peaks in VAF, which indicates two merged colonies. Samples with histograms like the middle or right were removed from the analysis.

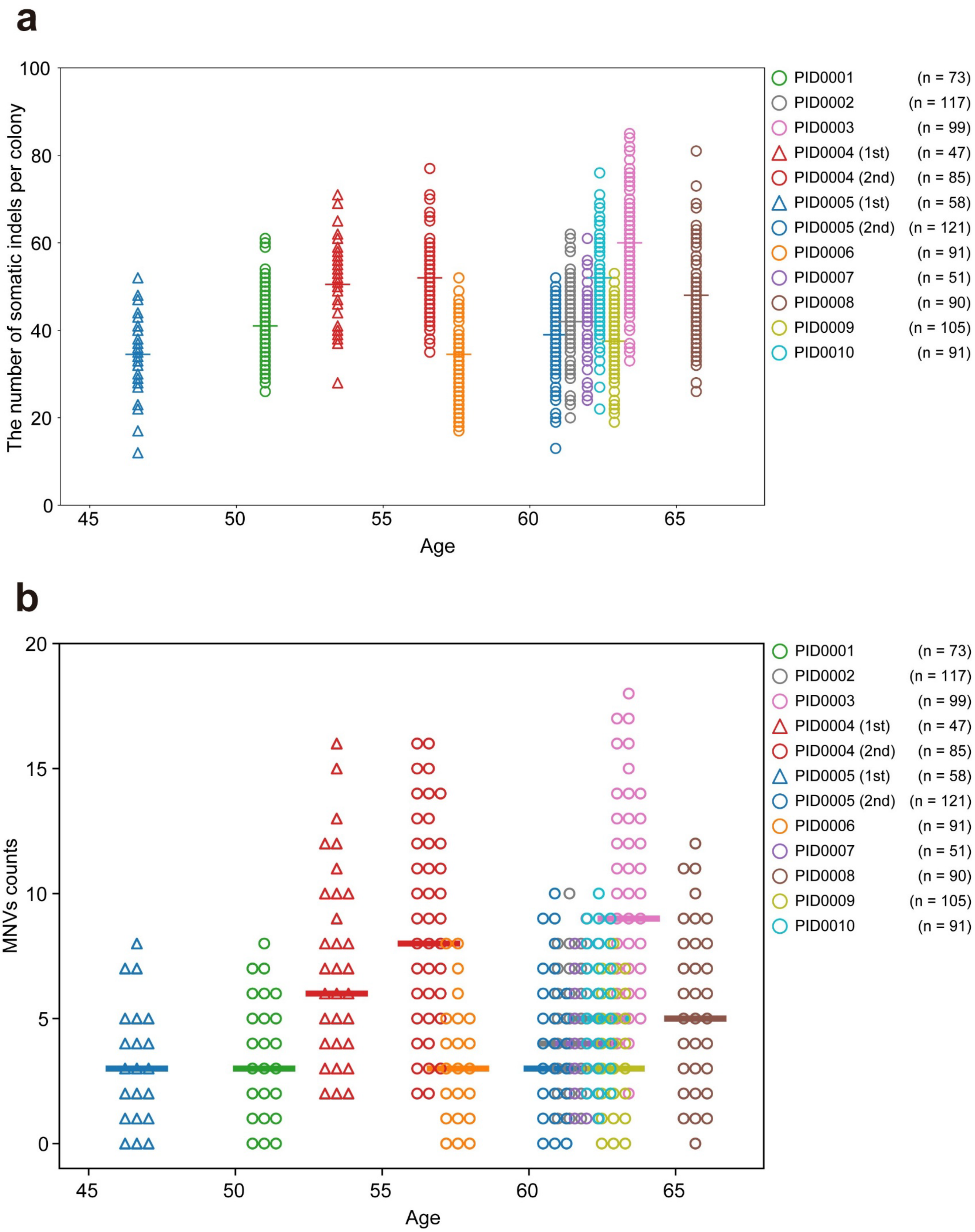
a
The number of somatic indels per colony
Age
b
MNVs counts
Age
PID0001 (n = 73)
PID0002 (n = 117)
PID0003 (n = 99)
PID0004 (1st) (n = 47)
PID0004 (2nd) (n = 85)
PID0005 (1st) (n = 58)
PID0005 (2nd) (n = 121)
PID0006 (n = 91)
PID0007 (n = 51)
PID0008 (n = 90)
PID0009 (n = 105)
PID0010 (n = 91)

Supplementary Figure 2. Age-related distribution of somatic indels and multiple nucleotide variants (MNVs) in HSPC colonies.

(**a**) Graph showing the correlation between the number of somatic indel variants in each colony and the age of the individual from whom the colony was derived. (**b**) Graph depicting the relationship between the number of somatic MNVs per colony and the age.

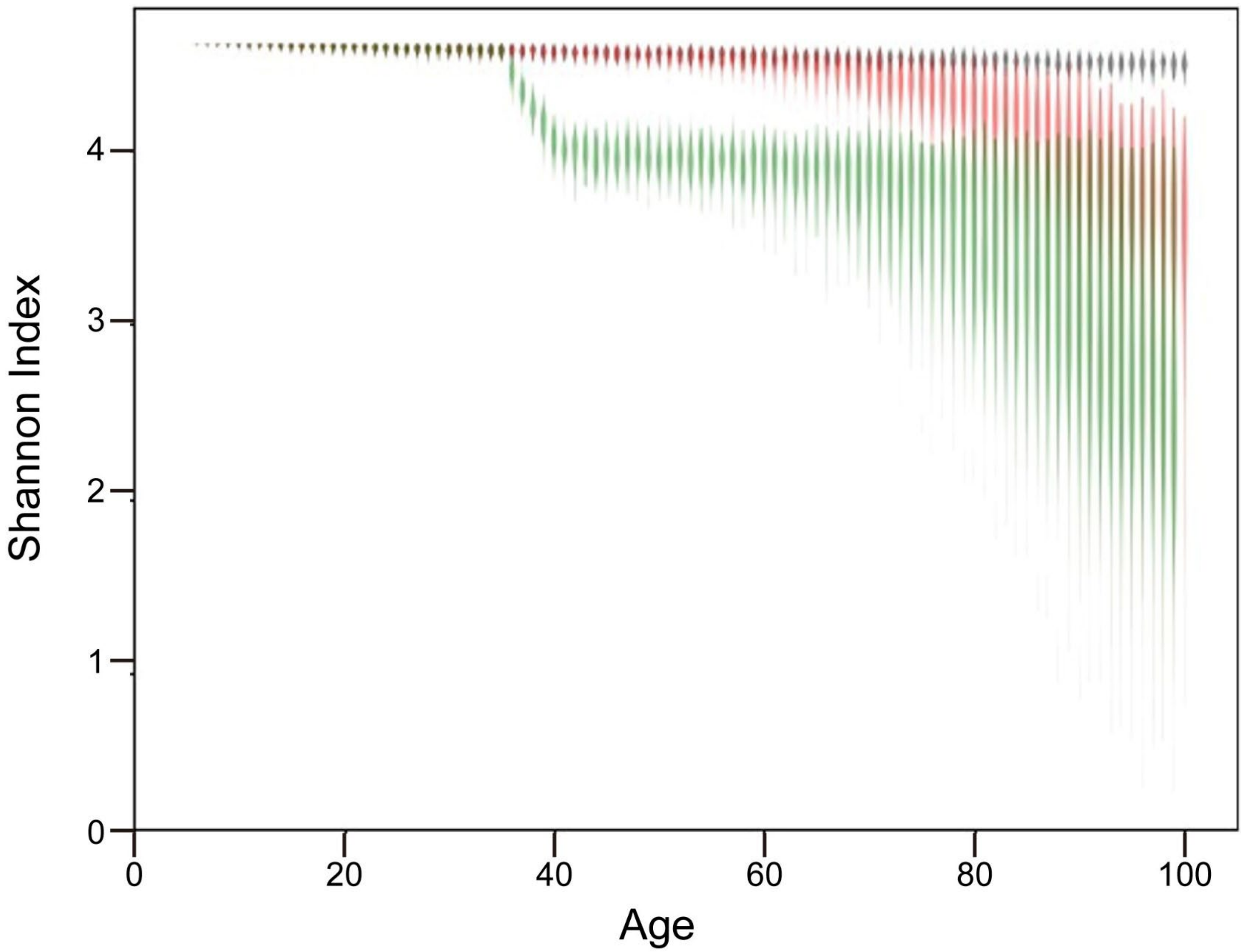

Supplementary Figure 3. Simulation of Shannon diversity index over time when 100 HSPCs are randomly sampled from 100,000 HSPC pool.
Black line assumes that acquired mutations have neutral fitness. Red line assumes that some mutations have selective advantage. Green line assumes that chemotherapy is administered around age 35 to 40.

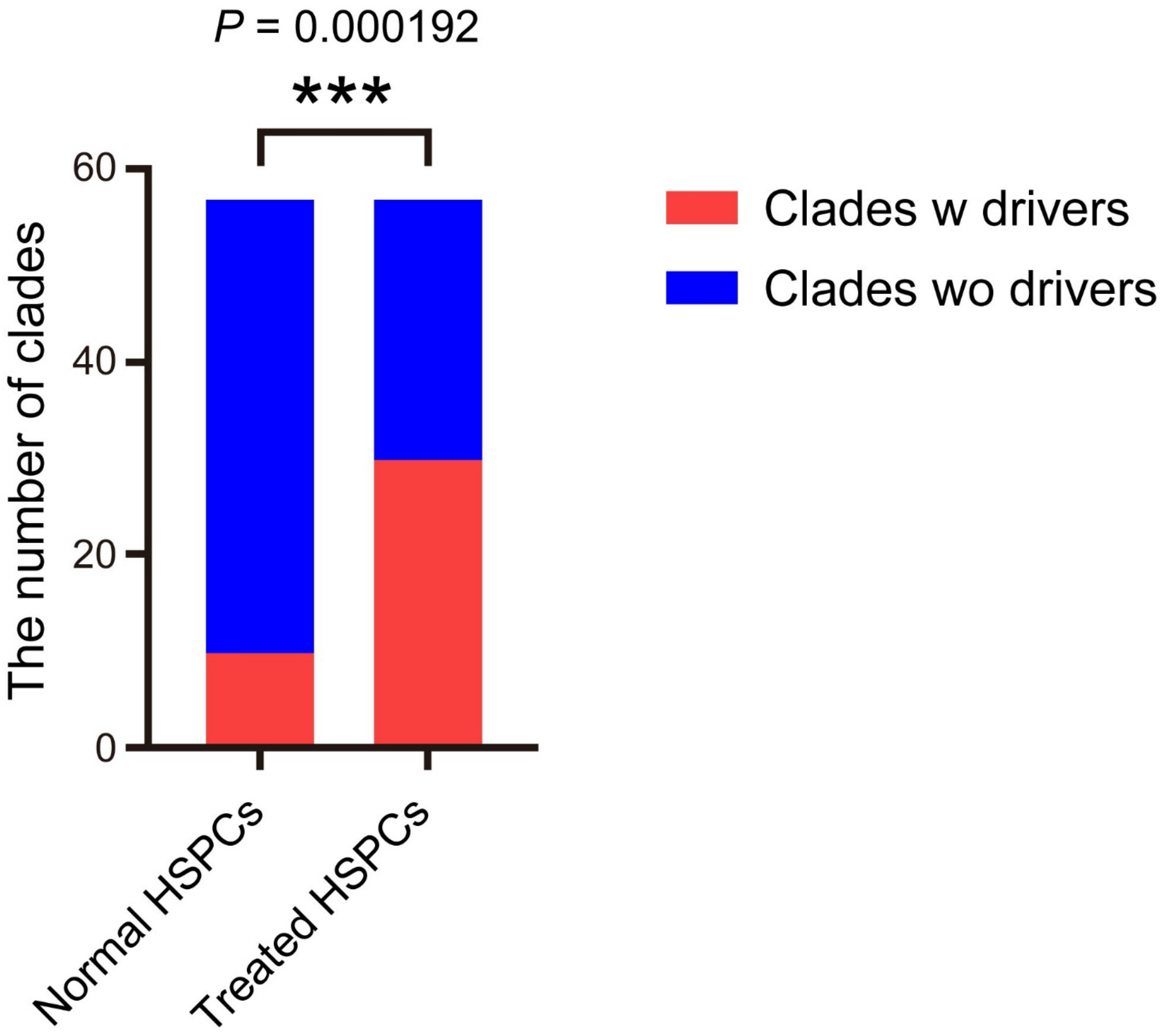

Supplementary Figure 4. Comparative analysis of clades harboring driver mutations in normal and treated hematopoietic stem and progenitor cells (HSPCs).
The graph contrasts the proportion of clades with driver mutations identified in a previously published dataset by Mitchell et al. against those found in the current study's chemotherapy-treated HSPCs. The statistical significance of the difference is denoted by ***, representing a P-value of less than 0.001, as determined by the two-sided Chi-Square test.

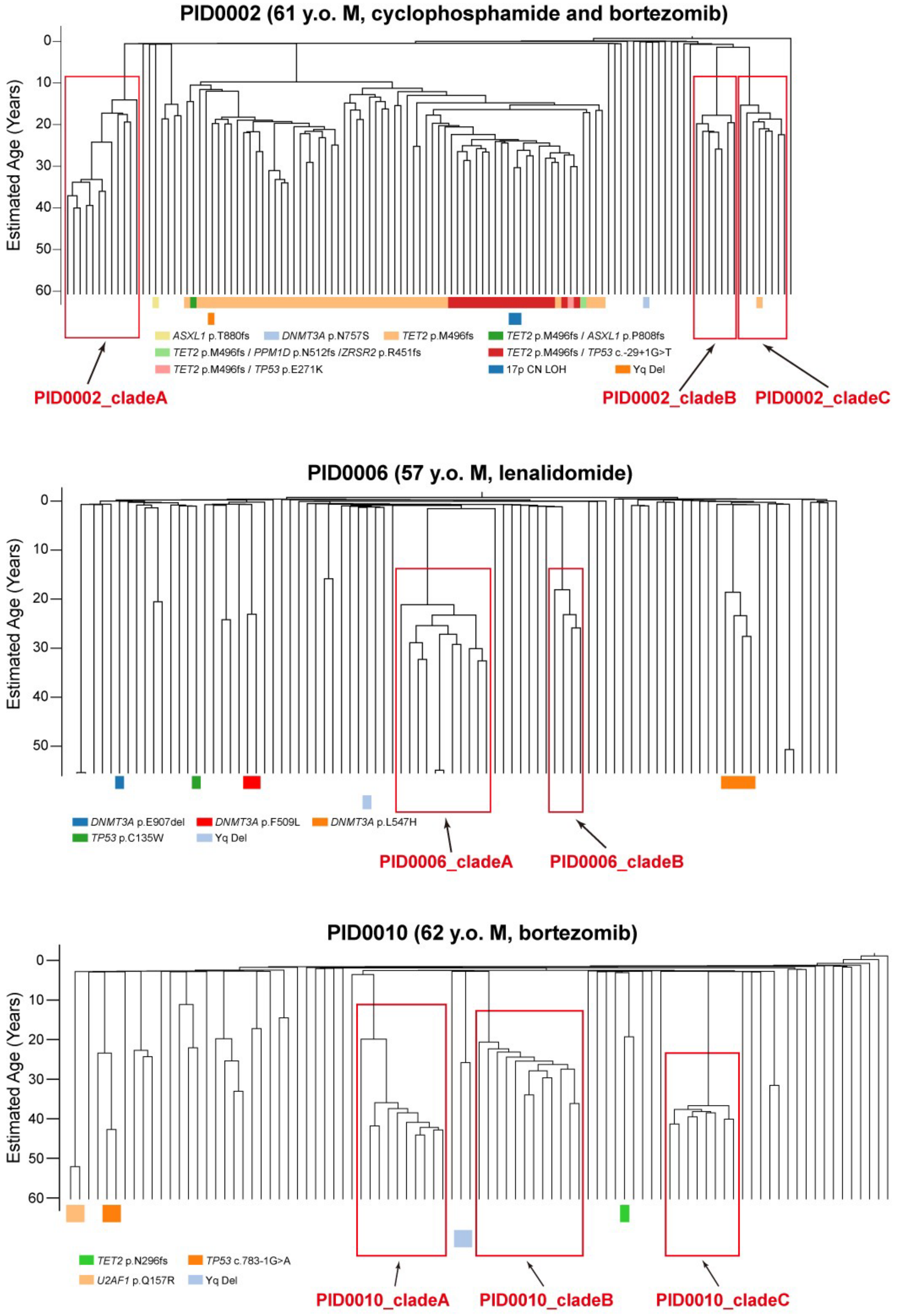

Supplementary Figure 5. The definition of clades without obvious driver mutations. These clades are used in the analysis of **Figure 5c** and **Figure 5d**.

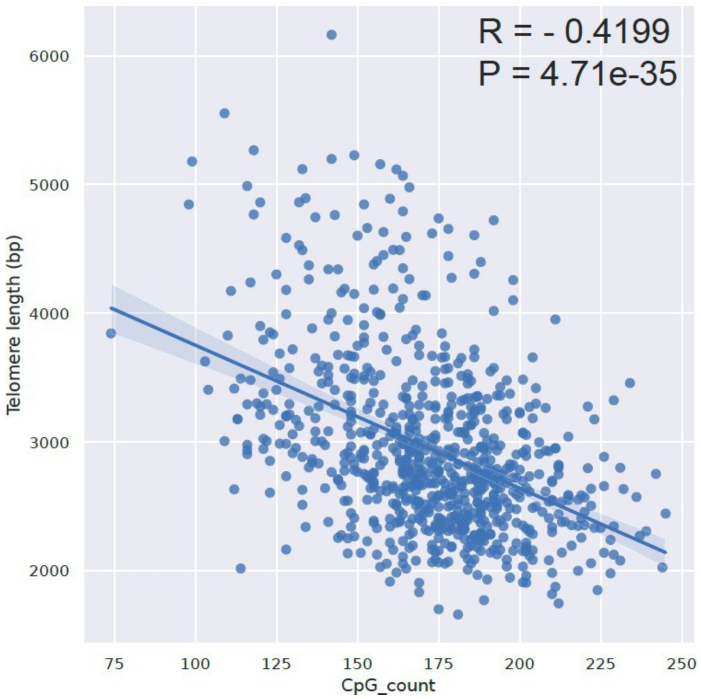

Supplementary Figure 6. Scatter plot correlating telomere length and C>T changes at CpG dinucleotides in each colony.

C>T changes at CpG sites are known to reflect cell division more accurately than the total mutation burden. Higher correlation coefficient was observed compared to the correlation with total mutation burden. Spearman correlation analysis was performed to assess the relationship between the count of C>T changes and the telomere length in each colony. The shaded region around the regression line indicates the 95% confidence interval for the regression estimate.

a

PID0002

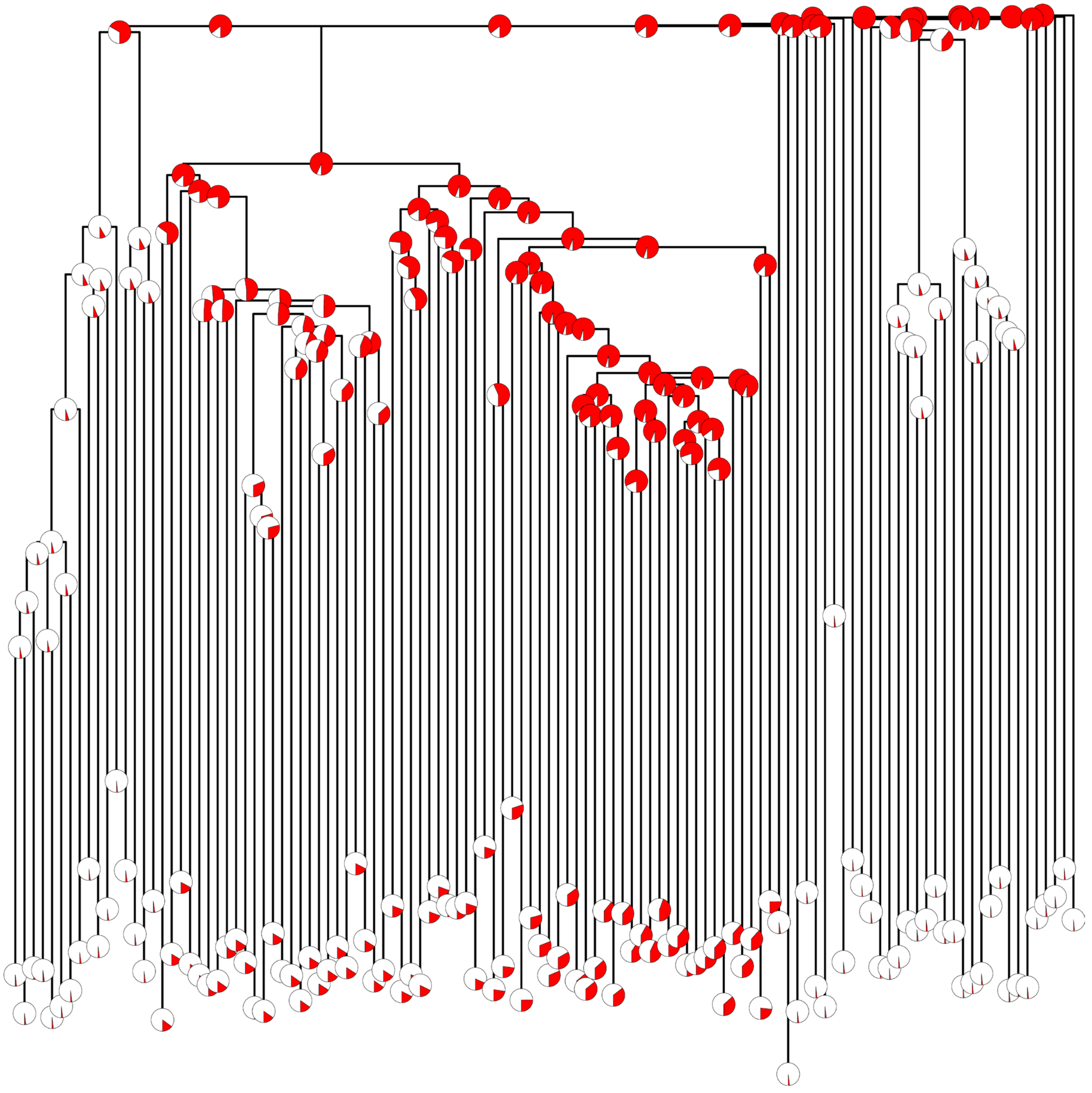

b

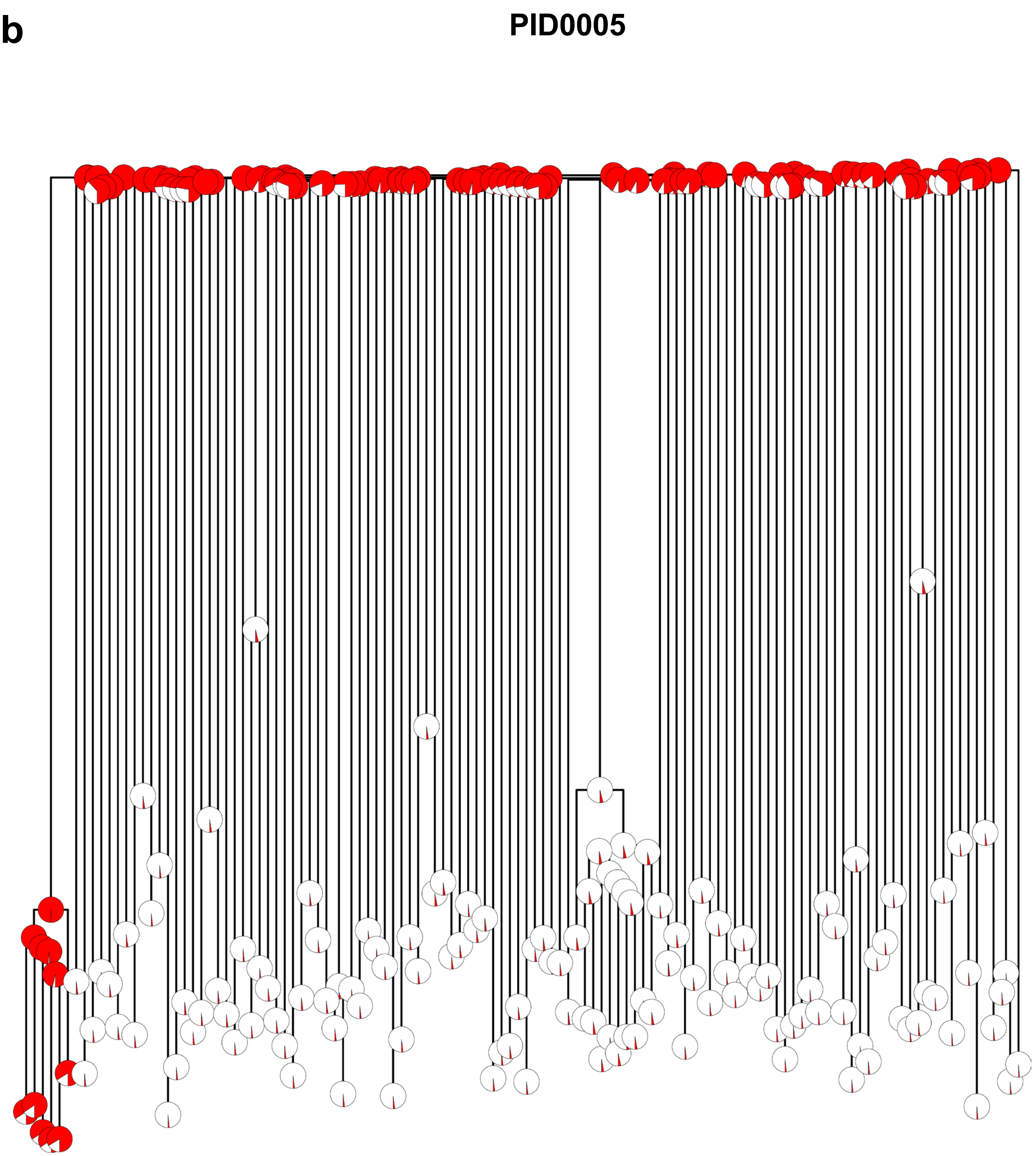

c

PID0006

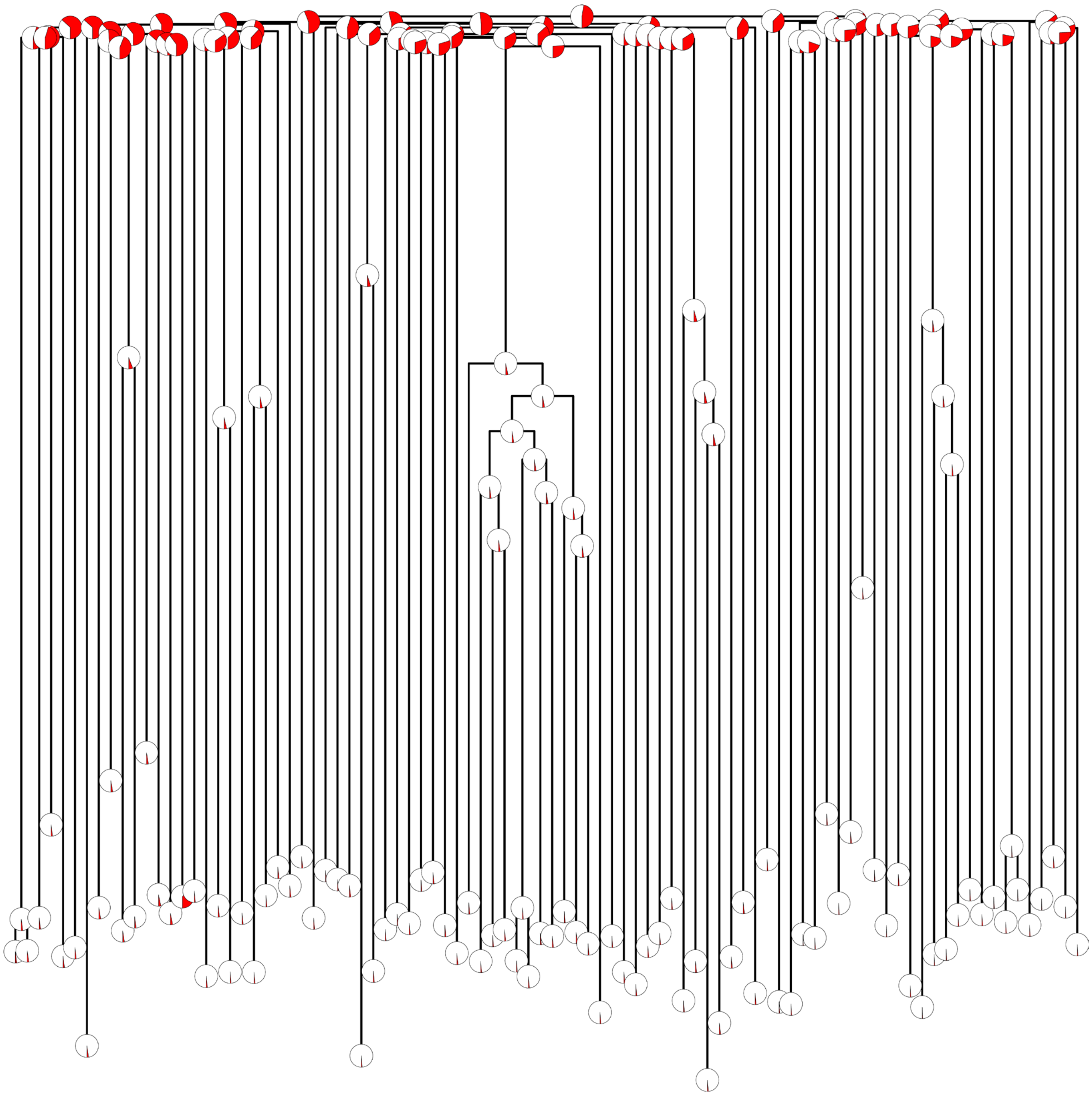

d

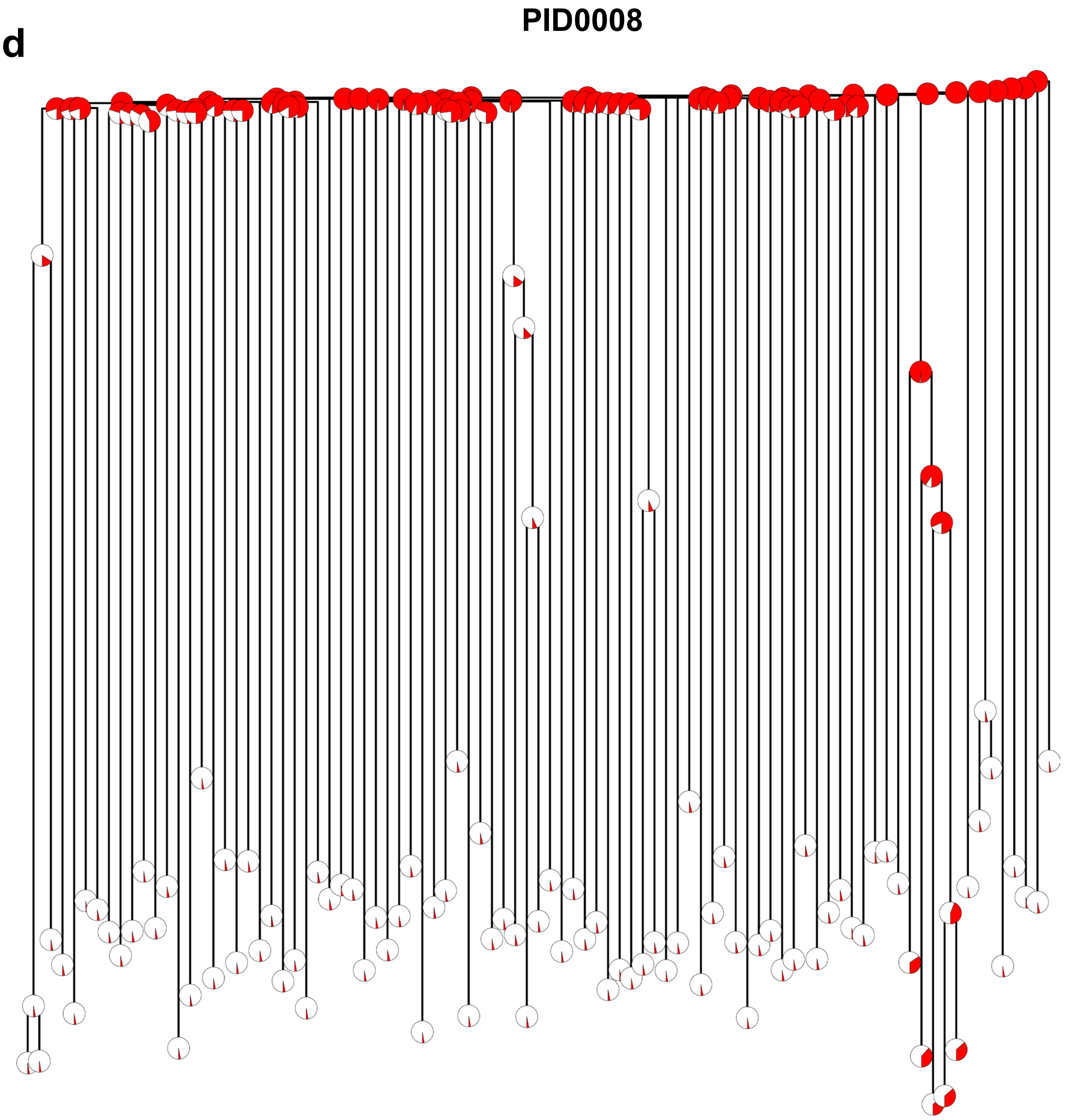

e

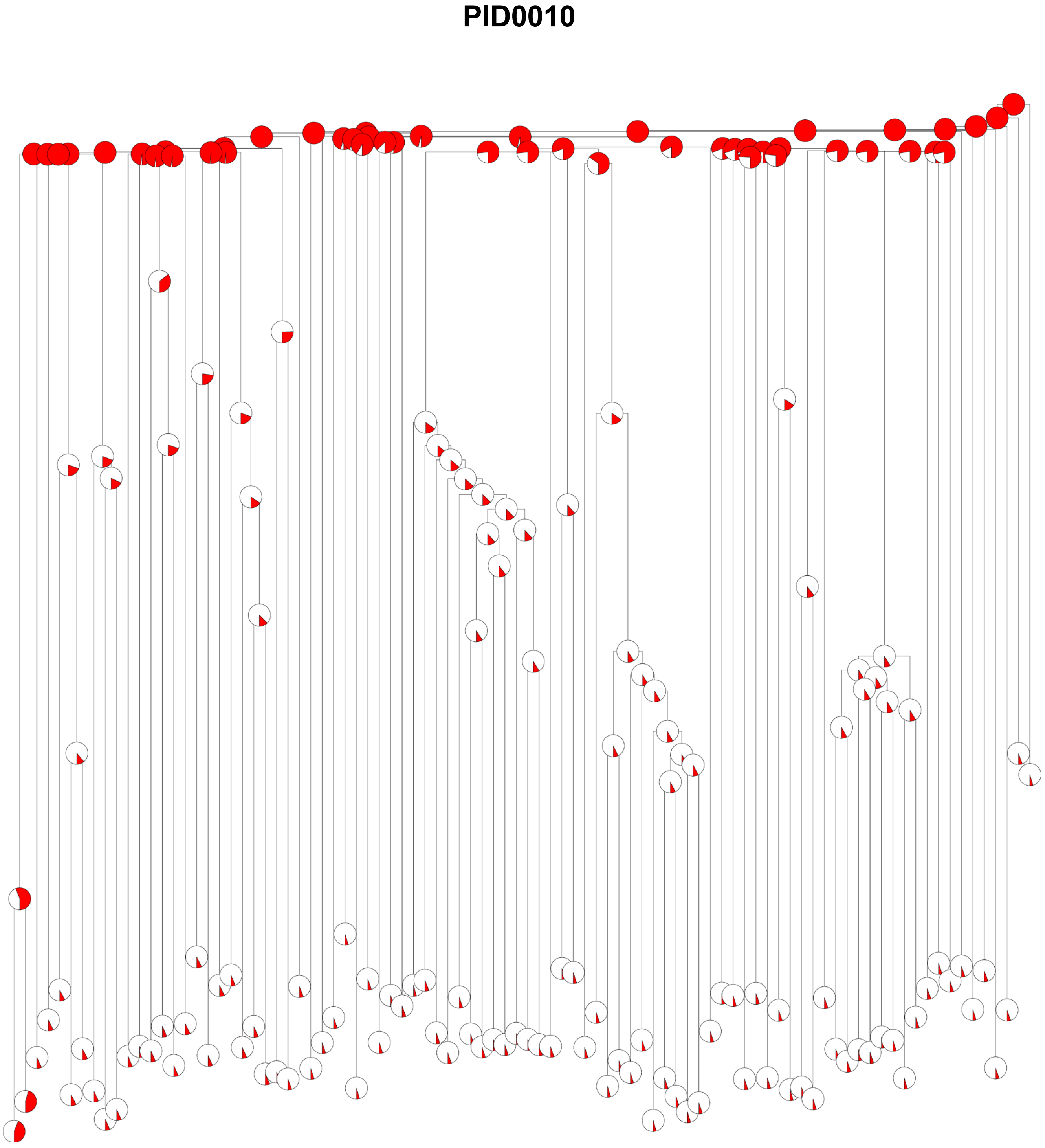

Supplementary Figure 7. Phylogenetic trees integrating HSPC colonies and matched t-MN genomes in samples where MRCA was identified.
The proportion of shared variants between individual colonies and t-MN samples are shown by the pie chart layered onto the trees. (**a**) PID0002, (**b**) PID0005, (**c**) PID0006, (**d**) PID0008, and (**e**) PID0010

a

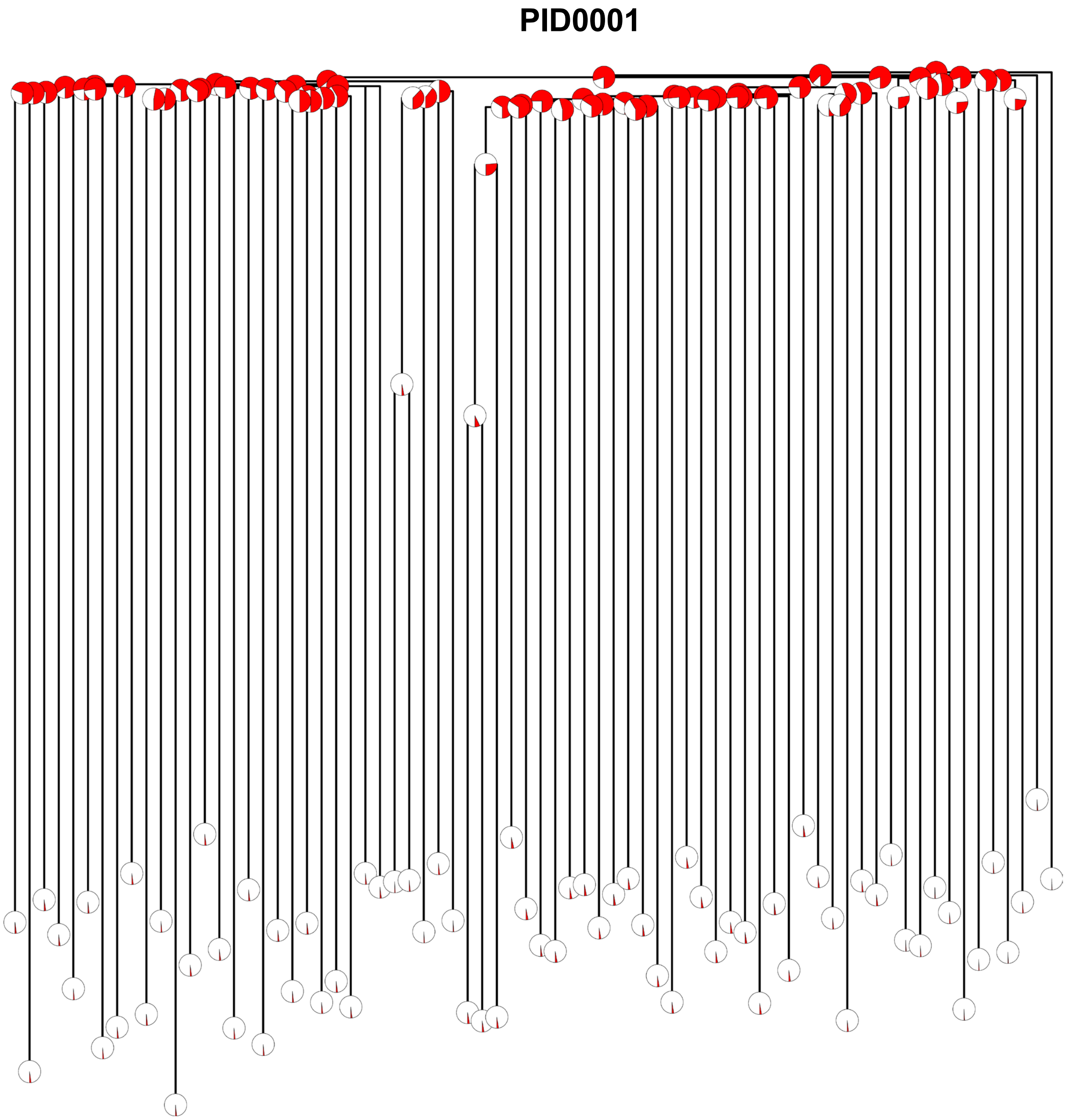

b

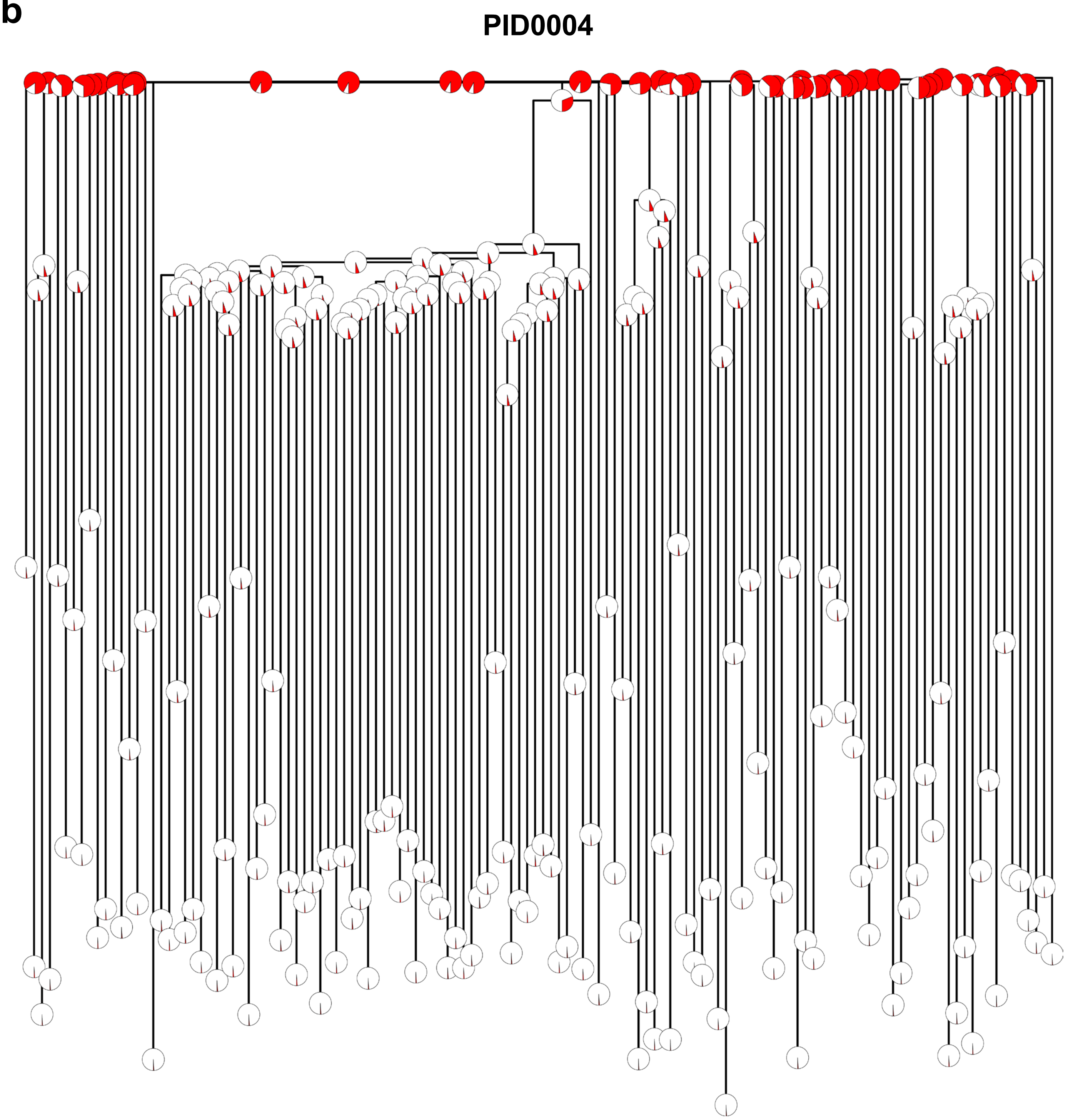

c

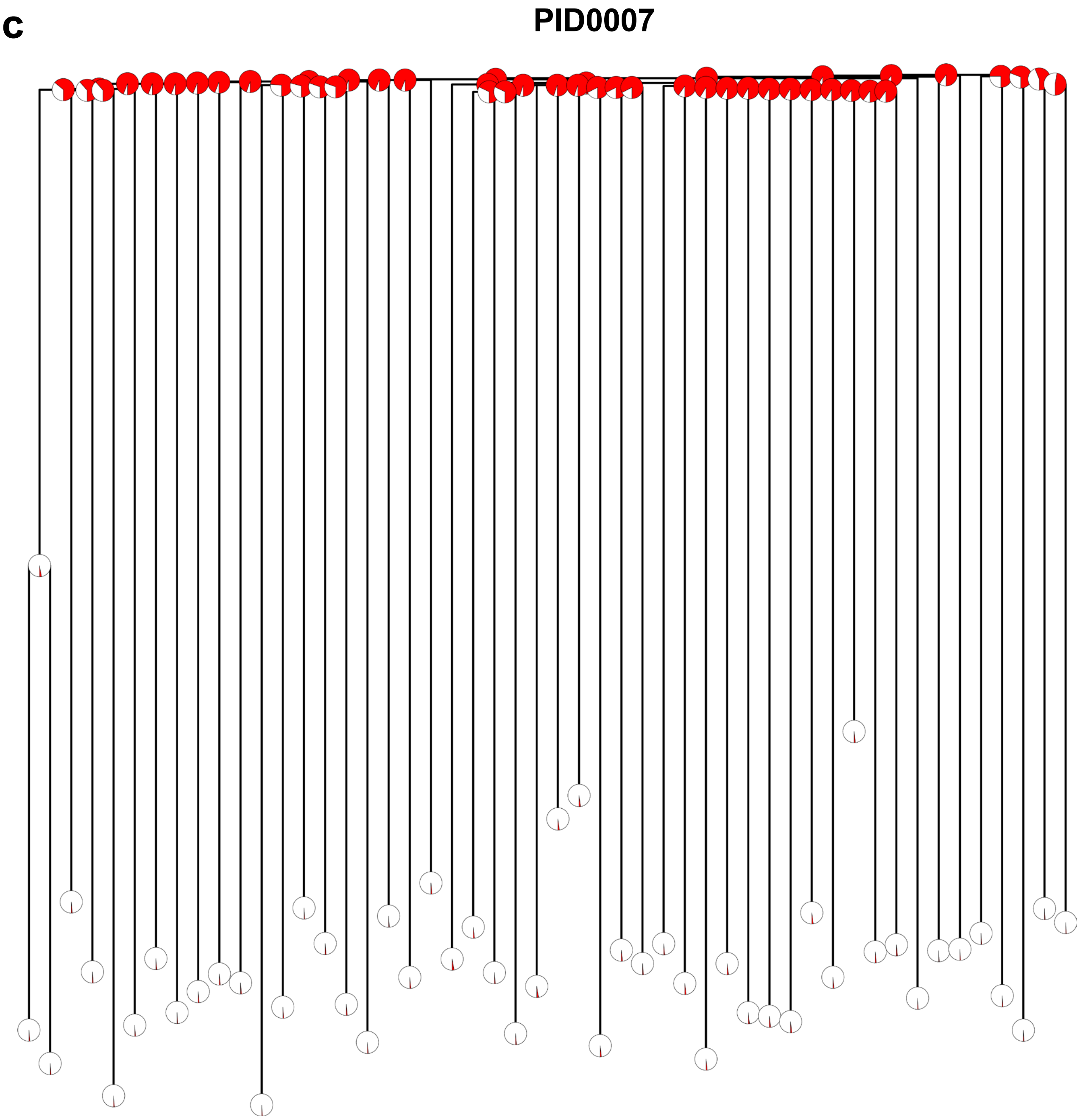

d

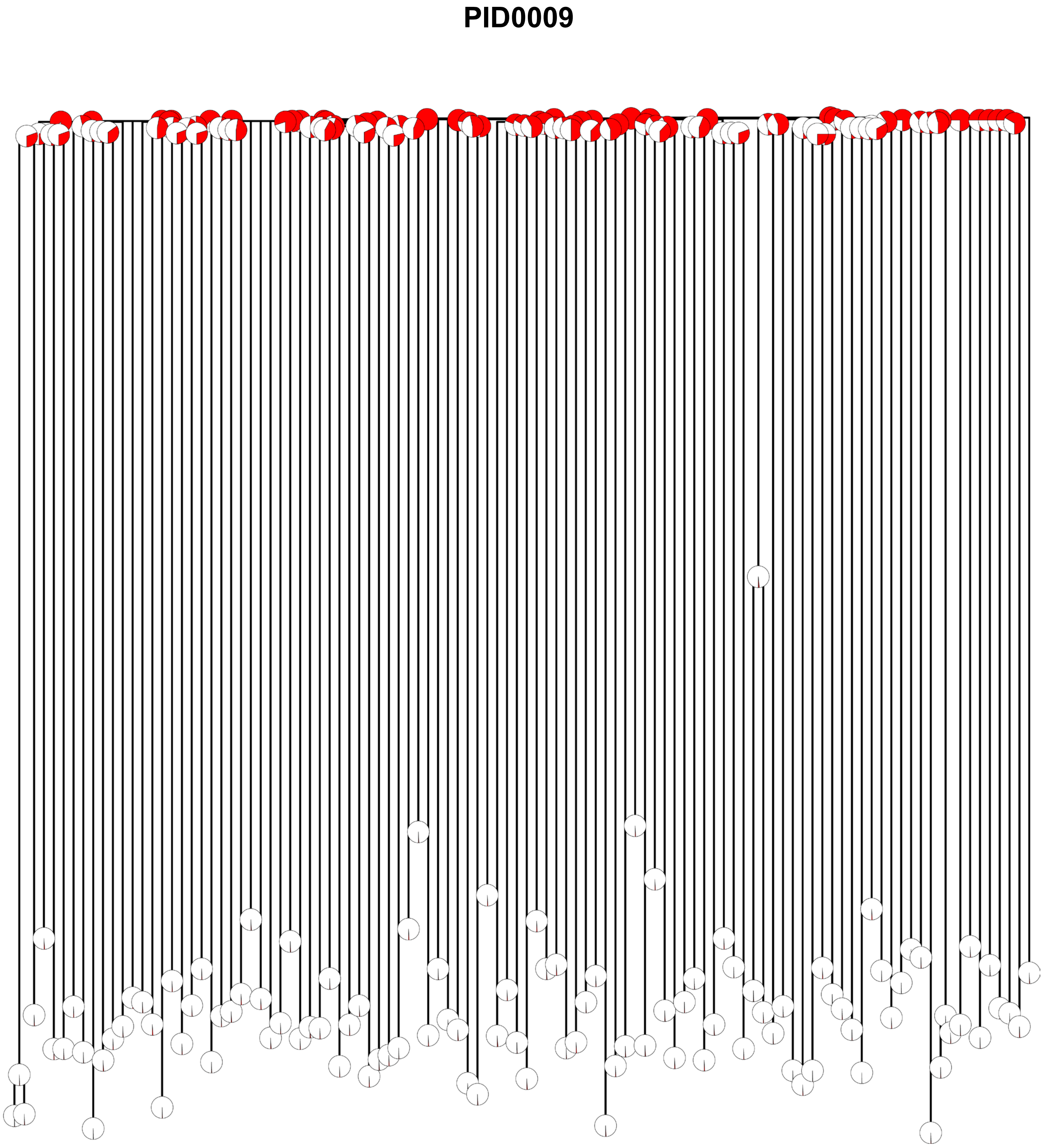

Supplementary Figure 8. Phylogenetic trees integrating HSPC colonies and matched t-MN genomes in samples where MRCA was not identified.
The proportion of shared variants between individual colonies and t-MN samples are shown by the pie chart layered onto the trees. (**a**) PID0001 (**b**) PID0004, (**c**) PID0007, and (**d**) PID0009
