# Supplementary material for: Clonal evolution of hematopoietic stem cells after autologous stem cell transplantation
Source: Nat Genet. 2025 Jul 1;57(7):1695–707. doi: 10.1038/s41588-025-02235-w (PMC12283406; doi:10.1038/s41588-025-02235-w)
Supplement: Supplementary file 2 — Reporting Summary [file 41588_2025_2235_MOESM2_ESM.pdf]

nature portfolio

Corresponding author(s): Koichi Takahashi, NG-A66064-R

Last updated by author(s): Mar 13, 2025

Reporting Summary

Nature Portfolio wishes to improve the reproducibility of the work that we publish. This form provides structure for consistency and transparency in reporting. For further information on Nature Portfolio policies, see our Editorial Policies and the Editorial Policy Checklist.

Statistics

For all statistical analyses, confirm that the following items are present in the figure legend, table legend, main text, or Methods section.

n/a	Confirmed	
☐	☒	The exact sample size (*n*) for each experimental group/condition, given as a discrete number and unit of measurement
☐	☒	A statement on whether measurements were taken from distinct samples or whether the same sample was measured repeatedly
☐	☒	The statistical test(s) used AND whether they are one- or two-sided<br>*Only common tests should be described solely by name; describe more complex techniques in the Methods section.*
☒	☐	A description of all covariates tested
☐	☒	A description of any assumptions or corrections, such as tests of normality and adjustment for multiple comparisons
☐	☒	A full description of the statistical parameters including central tendency (e.g. means) or other basic estimates (e.g. regression coefficient) AND variation (e.g. standard deviation) or associated estimates of uncertainty (e.g. confidence intervals)
☐	☒	For null hypothesis testing, the test statistic (e.g. *F*, *t*, *r*) with confidence intervals, effect sizes, degrees of freedom and *P* value noted<br>*Give P values as exact values whenever suitable.*
☒	☐	For Bayesian analysis, information on the choice of priors and Markov chain Monte Carlo settings
☒	☐	For hierarchical and complex designs, identification of the appropriate level for tests and full reporting of outcomes
☒	☐	Estimates of effect sizes (e.g. Cohen's *d*, Pearson's *r*), indicating how they were calculated

Our web collection on statistics for biologists contains articles on many of the points above.

Software and code

Policy information about availability of computer code

Data collection

No software was used for data collection.

Data analysis

Python 3.9.16
SigProfilerExtractor=1.1.21

R 4.1.0
ape=5.7.1
phangorn=2.11.1
phytolls=2.1.1
adephylo=1.1.16
mmsig=0.0.0.9000

Trim Galore, version 0.6.5
BWA Version: 0.7.17-r1198-dirty

The Genome Analysis Toolkit (GATK) v4.2.0.0
Picard Version: 2.23.8
PhyML version: 3.3.20211231
ANNOVAR version 2019.10.24

Other custom codes used to analyze the data in this study are available in the GitHub repository (https://github.com/Huryu-MDA/ClonalEvol_after_chemotherapy, https://github.com/KoichiSaeki/uryu-et-al-2025-moranmodel).

For manuscripts utilizing custom algorithms or software that are central to the research but not yet described in published literature, software must be made available to editors and reviewers. We strongly encourage code deposition in a community repository (e.g. GitHub). See the Nature Portfolio guidelines for submitting code & software for further information.

Data

Policy information about availability of data

All manuscripts must include a data availability statement. This statement should provide the following information, where applicable:

- Accession codes, unique identifiers, or web links for publicly available datasets
- A description of any restrictions on data availability
- For clinical datasets or third party data, please ensure that the statement adheres to our policy

Whole genome sequencing data from all colonies are available at Sequence Read Archive (SRA) with the project number PRJNA1058953 and PRJNA1206464. These are build with the human reference genome (GRCh37).

The following links are available for these SRA deposit data.

https://dataview.ncbi.nlm.nih.gov/object/PRJNA1058953?reviewer=d1vc57vpjnor7lsemigol66jd9
https://dataview.ncbi.nlm.nih.gov/object/PRJNA1206464?reviewer=bg7qbb137vjctq538c6pu4h345

Research involving human participants, their data, or biological material

Policy information about studies with human participants or human data. See also policy information about sex, gender (identity/presentation), and sexual orientation and race, ethnicity and racism.

Reporting on sex and gender	For all relevant analyses, the sex assigned at birth was utilized, including only those individuals whose genetically inferred sex corresponded with their sex assigned at birth.
Reporting on race, ethnicity, or other socially relevant groupings	This study does not assess the biological variations across different ethnic and racial groups.
Population characteristics	Patients with multiple myeloma who underwent autologous stem cell transplantation (ASCT) at The University of Texas MD Anderson Cancer Center were included in this study as sources for culturing single hematopoietic stem and progenitor cell (HSPC) colonies. In addition, we analyzed peripheral blood stem cells (PBSCs) from normal allogeneic donors as controls.The study cohort consisted of 10 multiple myeloma (MM) patients aged 46-65 years. These patients had been previously treated with various induction chemotherapies before undergoing PBSC collection for ASCT. Among them, two patients underwent ASCT twice, with PBSCs collected at two distinct time points (3 years and 15 years apart, respectively), both of which were analyzed in this study. The control group consisted of PBSCs from six healthy donors, aged 18-68 years, who had no history of chemotherapy exposure.<br>Treatment History<br>Prior therapy exposures for HSPCs included:<br>Melphalan (N=2), Cyclophosphamide (N=3), Doxorubicin (N=2), Vincristine (N=2), Lenalidomide or Thalidomide (N=7), Bortezomib (N=5), Radiation (N=2), Interferon alpha (N=1).<br><br>We sequenced a median of 89 colonies per sample (range: 46-128) for treated patients and 38 colonies per sample (range: 29-52) for normal donors, totaling 1,276 colonies (1,047 from treated patients and 229 from normal donors). Whole-genome sequencing (WGS) was performed at a median coverage of 29x (range: 14-68x).
Recruitment	Aliquots of cryopreserved peripheral blood stem cells (PBSCs) from multiple myeloma patients who underwent autologous stem cell transplantation (ASCT) were used to culture single HSPC colonies. As controls, cryopreserved PBSCs from healthy donors who had donated for allogeneic SCT were used. Written informed consent was obtained from all participating patients for sample collection and analysis.The cohort primarily consisted of patients previously treated with various induction chemotherapies, followed by PBSC collection and ASCT at our institution. To investigate the clonal evolution from normal HSPCs to t-MNs, we enriched the cohort with patients who subsequently developed t-MNs.
Ethics oversight	Written informed consent for the collection and analysis of samples was obtained from all participating patients. The study protocols were conducted in accordance with ethical guidelines and received approval from The University of Texas MD Anderson Cancer Center's institutional review board.

Note that full information on the approval of the study protocol must also be provided in the manuscript.

Field-specific reporting

Please select the one below that is the best fit for your research. If you are not sure, read the appropriate sections before making your selection.

☒ Life sciences ☐ Behavioural & social sciences ☐ Ecological, evolutionary & environmental sciences

For a reference copy of the document with all sections, see nature.com/documents/nr-reporting-summary-flat.pdf

Life sciences study design

All studies must disclose on these points even when the disclosure is negative.

Sample size	As the samples were obtained from cryopreserved specimens of myeloma patients, we utilized all available frozen stock from patients who were confirmed to have clonal hematopoiesis (CH) (variant allele frequency >2%) in their peripheral blood and subsequently developed therapy-related myeloid neoplasms (t-MN) following autologous hematopoietic stem cell transplantation (HSCT).<br>No formal sample size calculations were performed. However, we initially aimed to analyze at least 50 colonies per sample to find the CH driver mutations (VAF > 2%) from chemotherapy-treated patients. The actual number of colonies obtained varied due to limitations related to the availability of hematopoietic stem and progenitor cells in the graft. Consequently, we acquired a median of 89 colonies per sample (range: 46–128) for treated patients and 38 colonies per sample (range: 29–52) for normal donors.<br>According to our phylogenetic analysis, these sample numbers provided sufficient resolution to identify the majority of somatic driver mutations, trace the evolutionary trajectories of clonal expansions, and detect the most recent common ancestors (MRCAs) for the t-MN samples.
Data exclusions	We collected 1,276 single-cell-derived HSPC colonies from 10 multiple myeloma patients treated with chemotherapies and 6 normal donors. Colonies lacking a 50% VAF peak or displaying multiple peaks were excluded as likely merged colonies. Consequently, 1,261 colonies passed the quality control and were analyzed further.
Replication	We have ensured the reproducibility of our experimental findings through the following measures:<br><br>Detailed Protocols: We have provided comprehensive descriptions of our experimental protocols, allowing replication by other researchers.<br>Data Transparency: All relevant data have been made available in public repositories, ensuring accessibility for verification and further analysis.<br>Code Availability: The custom code utilized in our analyses has been shared openly, facilitating reproduction of our computational results.
Randomization	The analysis or study design was not randomized, as no therapy was administered as part of a clinical trial.
Blinding	The data analysis and collecting were not conducted in a blinded manner, and the investigators were aware of the allocation during both analysis and outcome evaluation.

Reporting for specific materials, systems and methods

We require information from authors about some types of materials, experimental systems and methods used in many studies. Here, indicate whether each material, system or method listed is relevant to your study. If you are not sure if a list item applies to your research, read the appropriate section before selecting a response.

Materials & experimental systems

n/a	Involved in the study	
☒	☐	Antibodies
☒	☐	Eukaryotic cell lines
☒	☐	Palaeontology and archaeology
☐	☒	Animals and other organisms
☒	☐	Clinical data
☒	☐	Dual use research of concern
☒	☐	Plants

Methods

n/a	Involved in the study	
☒	☐	ChIP-seq
☒	☐	Flow cytometry
☒	☐	MRI-based neuroimaging

Animals and other research organisms

Policy information about studies involving animals; ARRIVE guidelines recommended for reporting animal research, and Sex and Gender in Research

Laboratory animals	In our study, we utilized C57BL/6-based transgenic mice aged between 5 and 10 weeks at the start of the experiments.
Wild animals	The study did not involve wild animals.
Reporting on sex	In our study, we utilized donor bone marrow from age- and sex-matched 5- to 10-week-old Ppm1d R451X/+ (CD45.2), Trp53-/- (CD45.2), or wild-type control littermates. These were mixed with bone marrow from wild-type mice (CD45.1) in a 10:90 ratio, with a total of 3×10^6 cells transplanted into each recipient mouse. We ensured that both donor and recipient mice were matched for sex and age to maintain consistency and reliability in our experimental outcomes.<br><br>Sex-based analyses were not performed in this study, as the primary objective was to assess the effects of specific genetic modifications on hematopoietic stem cell function, irrespective of sex. Given our study's focus, we did not collect sex-disaggregated data.

Field-collected samples	The study did not involve samples collected from the field.
Ethics oversight	The study protocol involving mouse models was approved by the Institutional Animal Care and Use Committee (IACUC) of our institution (MD Anderson Cancer Center and/or Baylor College of Medicine).

Note that full information on the approval of the study protocol must also be provided in the manuscript.

Plants

Seed stocks	*Report on the source of all seed stocks or other plant material used. If applicable, state the seed stock centre and catalogue number. If plant specimens were collected from the field, describe the collection location, date and sampling procedures.*
Novel plant genotypes	*Describe the methods by which all novel plant genotypes were produced. This includes those generated by transgenic approaches, gene editing, chemical/radiation-based mutagenesis and hybridization. For transgenic lines, describe the transformation method, the number of independent lines analyzed and the generation upon which experiments were performed. For gene-edited lines, describe the editor used, the endogenous sequence targeted for editing, the targeting guide RNA sequence (if applicable) and how the editor was applied.*
Authentication	*Describe any authentication procedures for each seed stock used or novel genotype generated. Describe any experiments used to assess the effect of a mutation and, where applicable, how potential secondary effects (e.g. second site T-DNA insertions, mosiacism, off-target gene editing) were examined.*
